# Establishment and characterization of a HER2-enriched canine mammary cancerous myoepithelial cell line

**DOI:** 10.1186/s12917-023-03573-9

**Published:** 2023-01-30

**Authors:** Aolei Chen, Shaotang Ye, Jiahui Zheng, Jichao Li, Zejia Chen, Yashan Zhang, Shoujun Li

**Affiliations:** 1 College of Veterinary Medicine, South China Agricultural University, No.483 Wushanlu, Tianhe District, Guangzhou, Guangdong Province 510642 China; 2 Guangdong Provincial Key Laboratory of Prevention and Control for Severe Clinical Animal Diseases, No.483 Wushanlu, Tianhe District, Guangzhou, Guangdong Province 510642 China; 3 Guangdong Technological Engineering Research Center for Pet, No.483 Wushanlu, Tianhe District, Guangzhou, Guangdong Province 510642 China

**Keywords:** CMT, HER2, Myoepithelium, Mammosphere, EMT

## Abstract

**Background:**

Canine mammary tumors (CMTs) have a poor prognosis, along with tumor recurrence and metastasis. Cell lines are vital in vitro models for CMT research. Many CMT epithelial cell lines were reported. However, canine mammary myoepithelial cells, the contractile component of the canine mammary tissue were overlooked. This study aimed at establishing such a cell line. CMT-1 cell line was obtained from a canine mammary tumor CMT-1 and characterized molecularly through qPCR, western blotting, immunochemistry and immunofluorescence. Its doubling time, cytogenetic analysis and migration rate were evaluated using growth study, karyotype analysis and wound healing assay respectively. To determine its tumorigenesis, xenograft transplantation was performed.

**Results:**

CMT-1 tumor was a complex canine mammary carcinoma that stained negative to estrogen receptors (ER) and progesterone receptors (PR), but positive to human epidermal growth receptor-2 (HER2), defined as HER2-enriched subtype. In this study, a CMT-1 cell line obtained from CMT-1 tumor was immune-positive to vimentin, α-SMA, p63 and negative to E-cadherin (E-cad), indicating CMT-1 cells were myoepithelial cells. It was successfully cultured for more than 50 passages showing the same immunoreactivity to ER, PR, and HER2 as the primary canine tumor. The doubling time of CMT-1 cell line was 26.67 h. The chromosome number of CMT-1 cells ranged from 31 to 64. A potential spontaneous epithelial to mesenchymal transition (EMT) was noticed during cell cultures. Potential EMT-induced CMT-1 cells showed no significance in migration rate compared to the original CMT-1 cells. CMT-1 cells was able to grow on a 3D culture and formed grape-like, solid, and cystic mammospheres at different time period. Inoculation of CMT-1 cells induced a complex HER2-enriched mammary tumor with metastasis in mice.

**Conclusions:**

A canine cancerous HER2-enriched myoepithelial cell line was successfully established and a canine mammosphere developed from myoepithelial cells was documented in this study. We are expecting this novel cell line and its associated mammospheres could be used as a model to elucidate the role of myoepithelial cells in CMT carcinogensis in the future.

**Supplementary Information:**

The online version contains supplementary material available at 10.1186/s12917-023-03573-9.

## Background

Canine mammary tumors (CMT) are the most commonly seen neoplasm in intact female dogs, with approximately 50% being malignant [1]. Currently, the classification of CMT is based on its characteristics, such as morphology, tumor cell origins, and molecular markers [2–4]. However, subtle changes in mammary structure increase the difficulty of diagnosing CMT subtypes. To better identify tumor subtypes and obtain a more accurate diagnosis, immunohistochemistry (IHC) has gained popularity [4–6]. In comparative medicine, veterinary researchers have applied the immunophenotypes adopted from human breast cancer (HBC) to CMT using protein biomarkers such as estrogen receptor (ER), progesterone receptor (PR), and human epidermal growth factor receptor 2 (HER2). Based on the expression of the relevant biomarkers, the subtypes are classified as luminal A, luminal B, HER2-enriched, triple-negative, and basal-like [7]. The relationship between CMT immunophenotypes and prognostic significance has been extensively studied [5, 8, 9]

The *HER2* gene belongs to the epidermal growth factor receptor (EGFR) family, activation of which is associated with increased proliferation, angiogenesis, invasiveness, and epithelial-mesenchymal transition (EMT) [10, 11]. Approximately 15–20% of the HBC is classified as HER2 positive with HER2-enriched being the most common subgroup, which is associated with a worse prognosis [12]. On the other hand, the prevalence of HER2-positive CMT is 8.3–22% [9, 13]. It was reported that overexpression of HER2 protein was positively associated with nuclear pleomorphism, histological grade and mitotic index in CMT, suggesting *HER2* gene as an oncogene [14, 15]. While other argued that the expression of HER2 protein may not correlate to metastasis and demonstrated no prognostic relevance [16, 17]. Whether *HER2* gene in CMT could be used as a prognostic and predictive factor as it is in HBC require further research evidence.

The mammary gland is composed of an epithelial cell lineage and a myoepithelial (ME) cell lineage. ME cells are contractile cells that express α-smooth muscle actin (α-SMA) and are arranged in a continuous monolayer in the mammary duct and appear in a stellate shape around the mammary acini [18]. Recently, ME cells have captured some attention owing to their potential tumor-suppressive effect [19].

In summary, our study showed a successful establishment of a HER2-enriched canine mammary myoepithelial cell line and examined its morphology, growth pattern, karyotype, and protein expression. This study also exhibited different morphologies of canine mammospheres formed by homogenous canine myoepithelial cells over time.

## Results

### Establishment of the CMT-1 cell line from a malignant HER2-enriched CMT

**Fig. 1:**
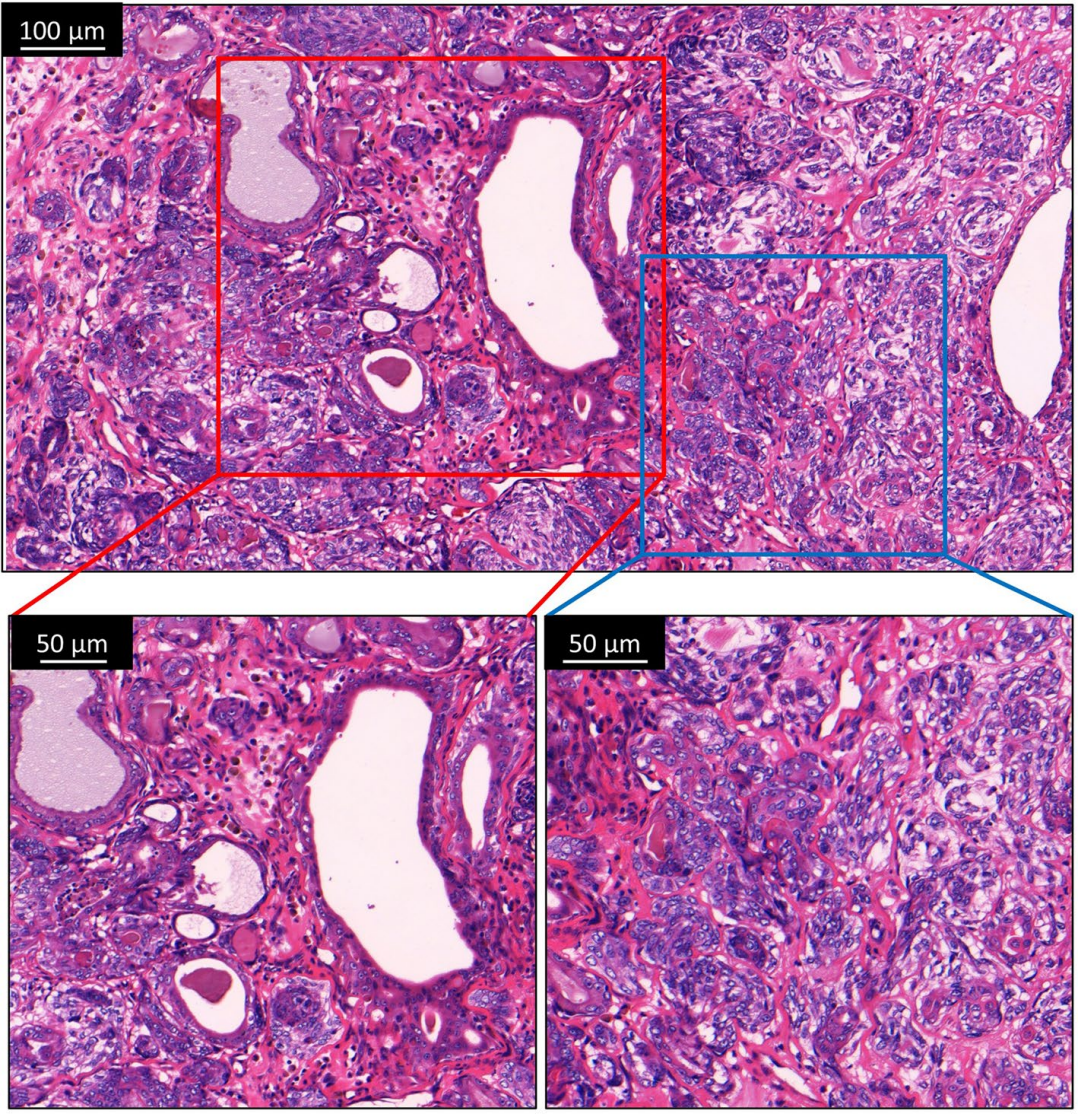
Histopathology of the CMT-1 tumor with H&E and IHC staining. Lower left: Uncapsulated mass composed of proliferative mammary epithelial and myoepithelial cells. Lower right: Nuclei were oval to elongated containing finely stippled scant chromatin and indistinct nucleoli

**Fig. 2:**
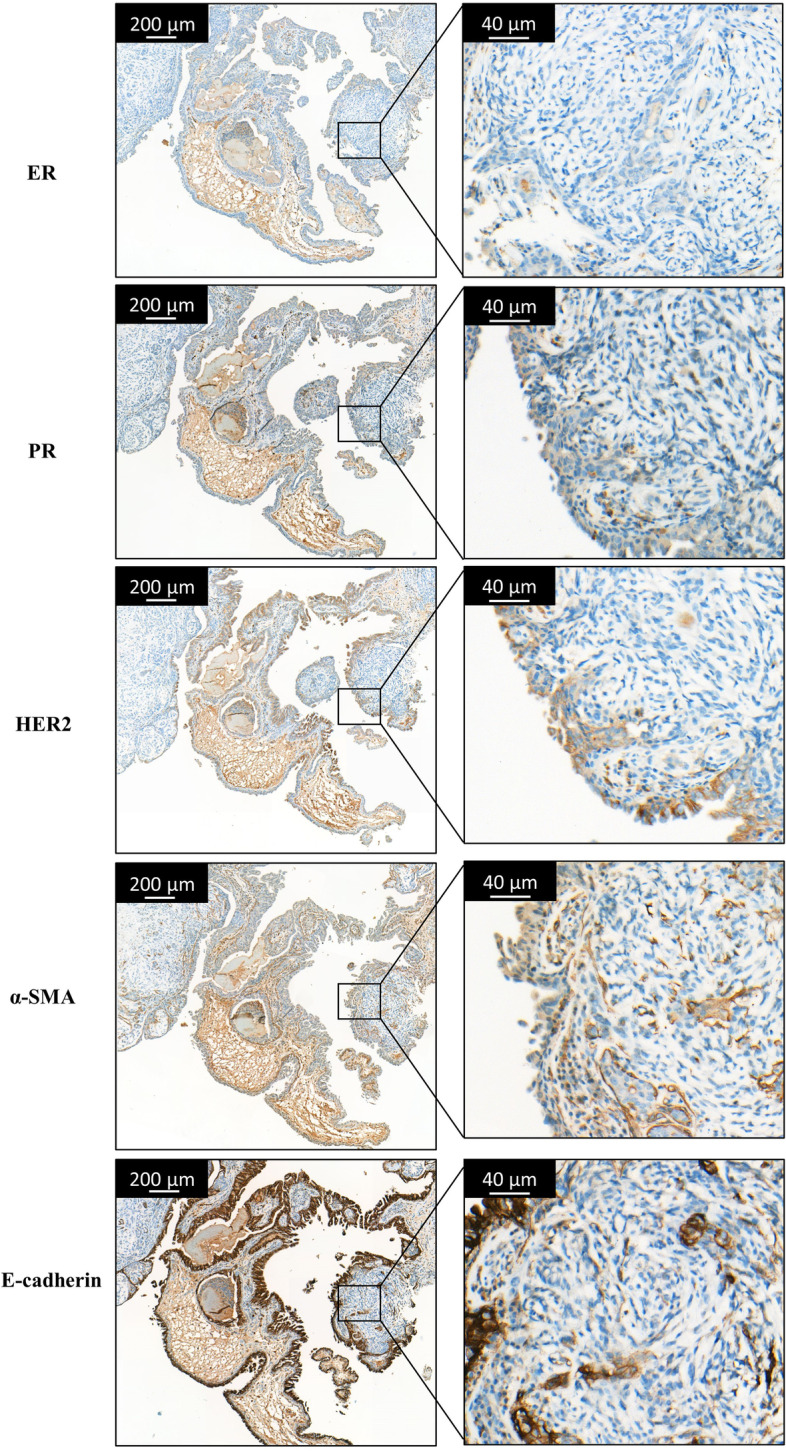
Histopathology of the CMT-1 tumor with IHC staining. IHC staining images showed that CMT was ER negative, PR negative, HER2 positive, α-SMA positive, and E-cadherin positive

The primary cell culture was obtained from a 10-year-old female intact Chihuahua that was presented to the South China Agricultural University Veterinary Teaching Hospital for a mammary mass. The canine mammary tumor was diagnosed based on its signalment, history, and clinical findings. No metastasis was noted on the thoracic radiographs and abdominal ultrasound. Standard mastectomy was performed with informed consent obtained from the owner. Histopathology revealed that mammary epithelial cells formed small tubular structures and neoplastic myoepithelial cells were spindoloid and located in the extracellular matrix. There was moderate anisocytosis and anisokaryosis. Rare mitotic figures were seen (Fig. 1). This is consistent with complex low grade canine mammary carcinoma. IHC detected no expression of ER and PR, moderate expression of HER2, and strong expression of E-cadherin in the primary tumor (Fig. 2). According to the Herceptest™ interpretation manual, CMT-1 was categorized as HER2-enriched (2 +) subtype.

### Growth studies and doubling time of the CMT-1 cell line

**Fig. 3:**
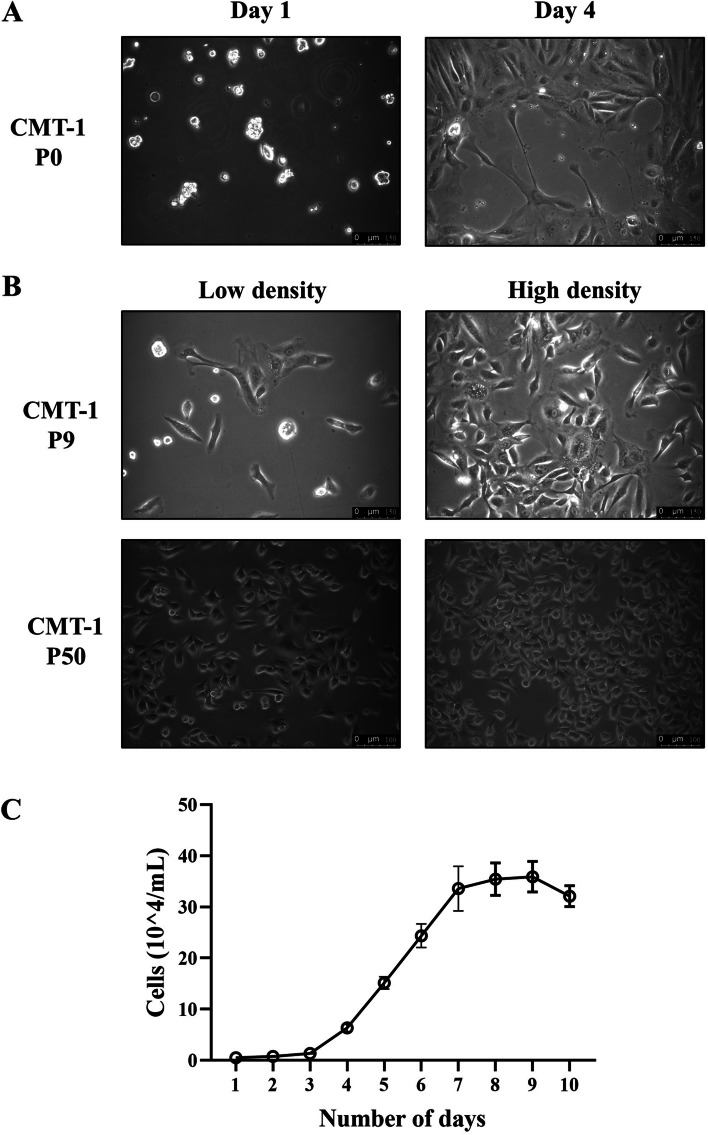
Growth studies and doubling time of the CMT-1 cell line. **A** CMT-1 cells demonstrated moderate anisocytosis and anisokaryosis with the majority of cells having multiple nuclei. **B** CMT-1 cells (passage 9) showed slight anisocytosis and anisokaryosis with the majority of cells having a single nucleus while CMT-1 (passage 50) became more uniformly round to oval in morphology. **C** The population doubling time of CMT-1 (passage 50) was calculated to be 26.67 h

In order to investigate the growth property and morphology of the CMT-1 cell line, we observed the shape of CMT-1 cells during cultivation passages and performed a growth study assay. CMT-1 cells were polygonal cells with, initially, multiple nuclei in one cell (passage 9). With cell passage number increased, the CMT-1 cells became less spindoloid and tended to be uniformly round to oval shape at passage 50 (Fig. 3A-B). This may indicate that CMT-1 cells became more stable in characteristics allowing for further study. Growth studies showed that the doubling time of the cell line (passage 50) was 26.67 h (Fig. 3C).

### Karyotype Analysis of the CMT-1 cell line

**Fig. 4:**
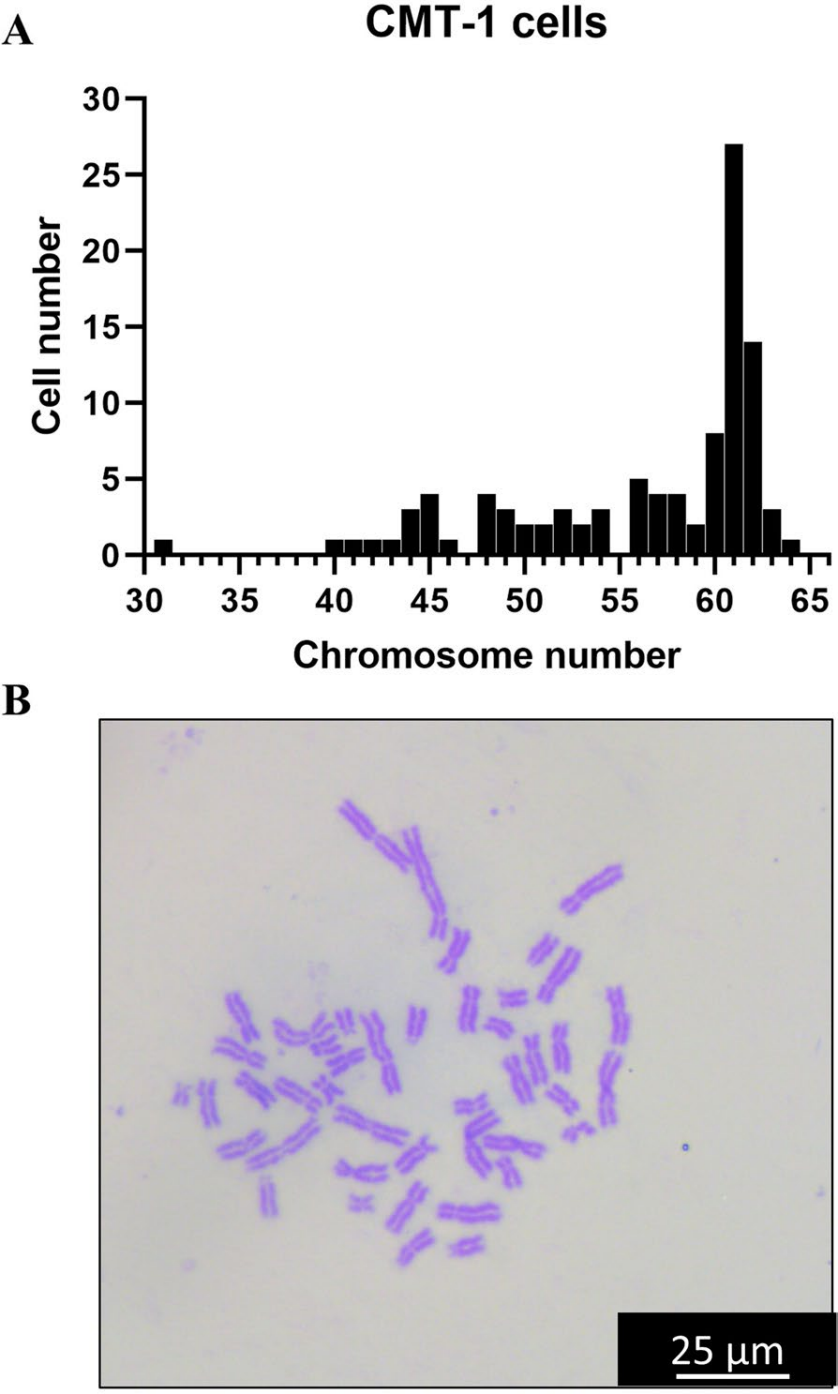
Karyotype analysis of the CMT-1 cell line. **A** The modal number of chromosome number in CMT-1 cell line was 61. **B** Chromosomal structural abnormalities such as gaps, breaks and fragments were noted

100 cells with evenly distributed chromosomes were chosen for observation and analysis. CMT-1 cells showed both numerical and structural abnormalities. The chromosome number of CMT-1 cells ranged from 31 to 64 while the normal number is 78 in canine (Fig. 4A). Chromosomal structural chromosomal abnormalities such as gaps, breaks and fragments were noted (Fig. 4B).

### CMT-1 cells were HER2-enriched myoepithelium

**Fig. 5:**
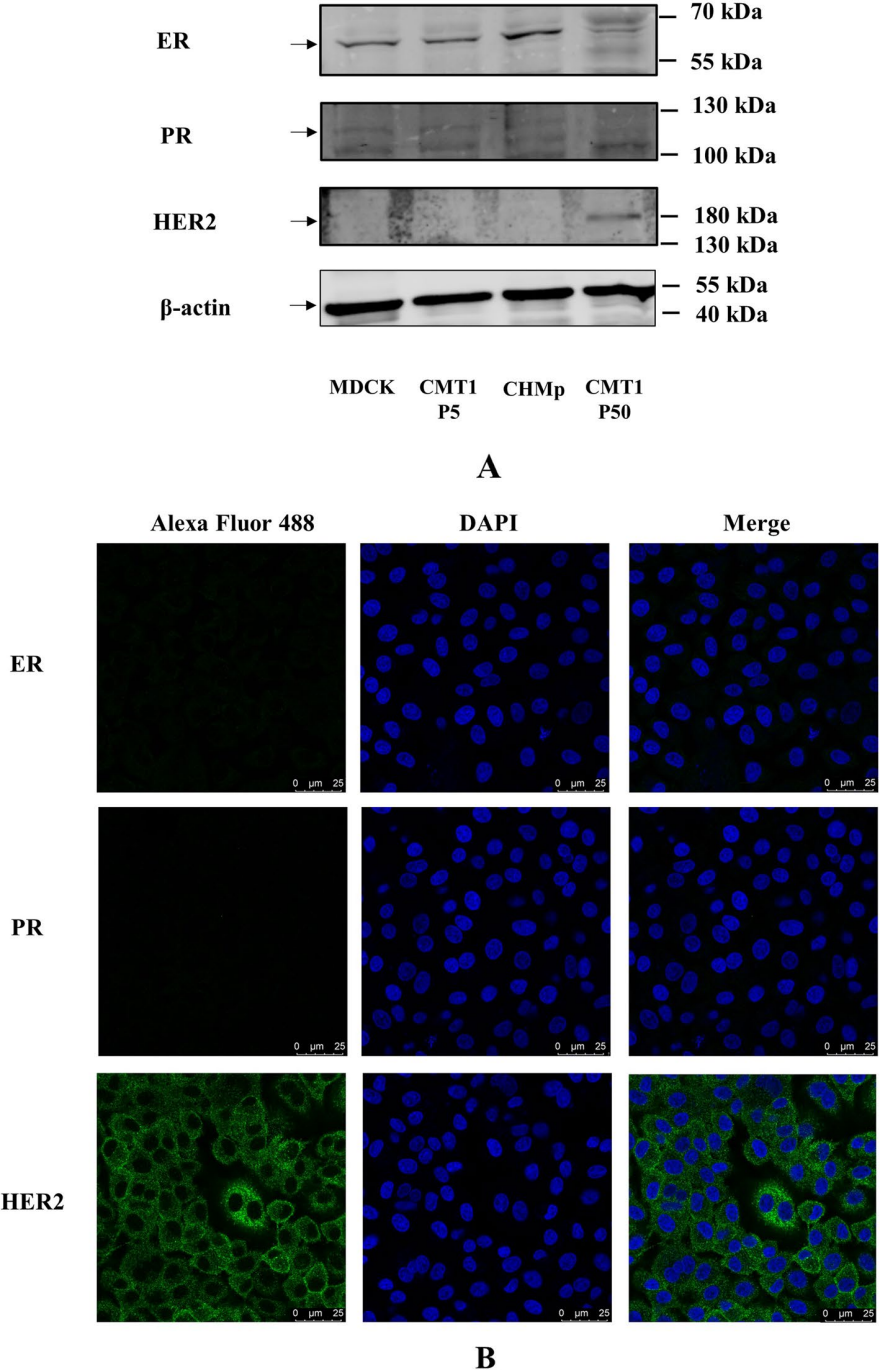
Western blotting results of different cell lines and immunofluorescence assay results of CMT-1 cells. **A** The results of western blotting of MDCK cells, CMT-1 cells (passage 5), CHMp cell line, and CMT-1 cells (passage 50) were ER^+^/PR^−^/HER2^−^, ER^+^/PR^−^/HER2^−^, ER^+^/PR^−^/HER2^−^ and ER^−^/PR^−^/HER2.^+^ respectively. **B** IFA results of CMT-1 cells were consistent with WB results. The original images of the western blot were shown in the Supplementary file 1

**Fig. 6:**
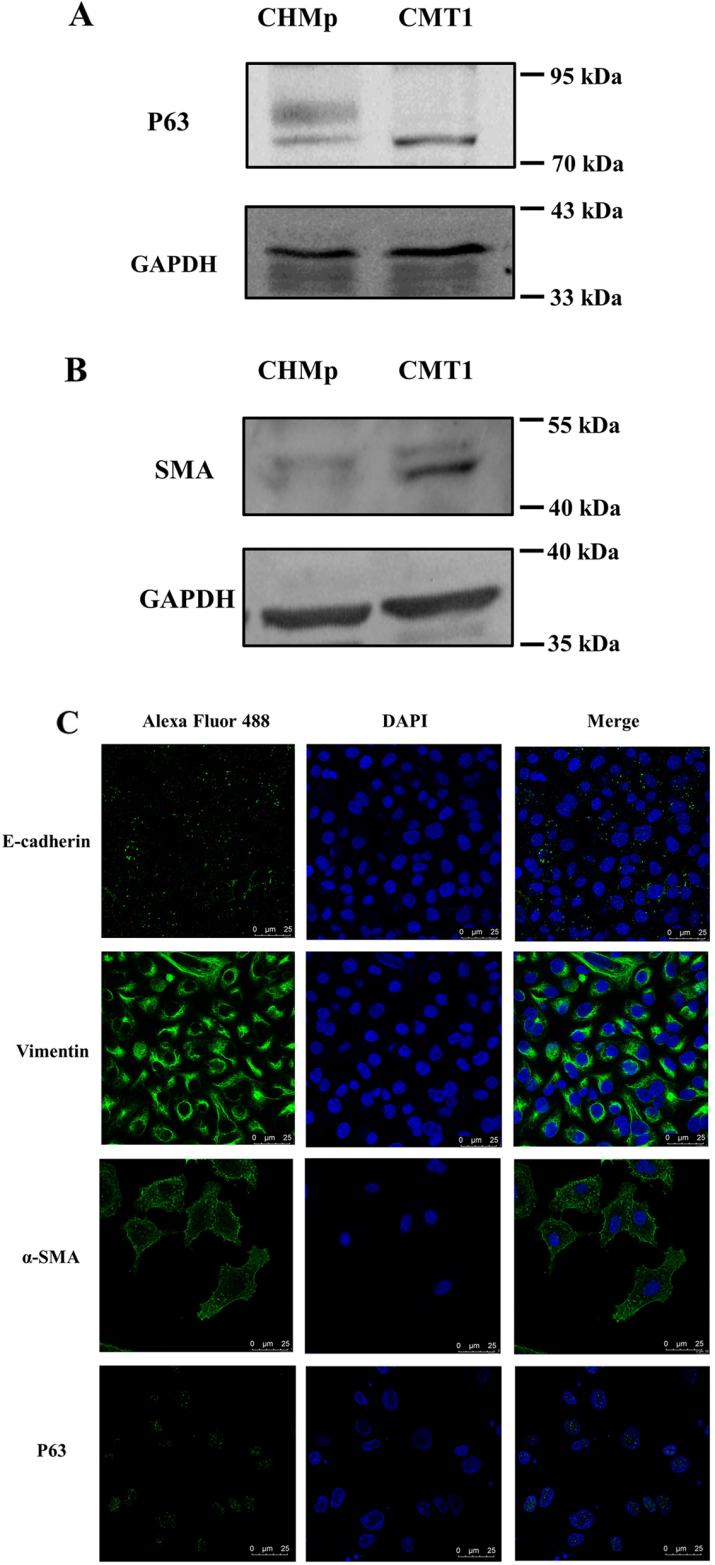
Molecular analysis of the CMT-1 cell line. **A**&**B** The results of western blotting showed CMT-1 cells were positive to α-SMA and p63. **C** IFA results demonstrated CMT-1 cells had weak reaction to E-cadherin and moderate to strong reaction to vimentin. IFA results shared the same immunoreactivity to α-SMA and p63 for CMT-1 cells. The original images of the western blot were shown in the Supplementary file 1

To identify which subgroup the CMT-1 cells belonged to, we detected the expression of ER, PR, and HER2 using WB and IFA. The expression of E-cadherin, vimentin, α-SMA and p63 was also detected by WB and IFA to determine the cell lineage of CMT-1 cells. Morphologically, CMT-1 cells were spindle-shaped under a light microscope (Fig. 3A). Molecularly, CMT-1 cells (passage 50) were negative for ER and PR but positive for HER2 in WB assays (Fig. 5A). MDCK cells were used as a positive control for ER and negative control for PR and HER2. Compared to MDCK cells, the CMT-1 cell line was negative for ER and PR but positive for HER2. Interestingly, as the CMT-1 cell passage increased, the CMT-1 cell line lost ER expression but gained HER2 expression by passage 50. Uyama et al. confirmed that CHMp cells, another canine mammary tumor cell line, were positive for E-cadherin using flow cytometry but their hormonal receptors and HER2 status have not been evaluated [20]. In this study, we detected moderate ER expression, weak PR expression, and no HER2 expression of CHMp cells. Hence, CHMp cells were categorized as a luminal subtype. As for myoepithelial cell markers, CMT-1 cells were positive to both α-SMA and p63 (Fig. 6A-B). In IF images, although a scant amount of ER and PR signaling was received, given the location of the signals, CMT-1 cells were considered ER and PR negative [21]. Meanwhile, CMT-1 cells showed a moderate to strong signal of HER2 (Fig. 5B). Moreover, the CMT-1 cell line demonstrated weak reaction to E-cadherin but a moderate to strong reaction to vimentin, α-SMA and weak to moderate reaction to p63 in the cytoplasm, cytomembrane and nucleus, respectively (Fig. 6C). Therefore, we defined CMT-1 cells as HER2-enriched myoepithelial cells.

### Potential epithelial-mesenchymal transition property of the CMT-1 cells

**Fig. 7:**
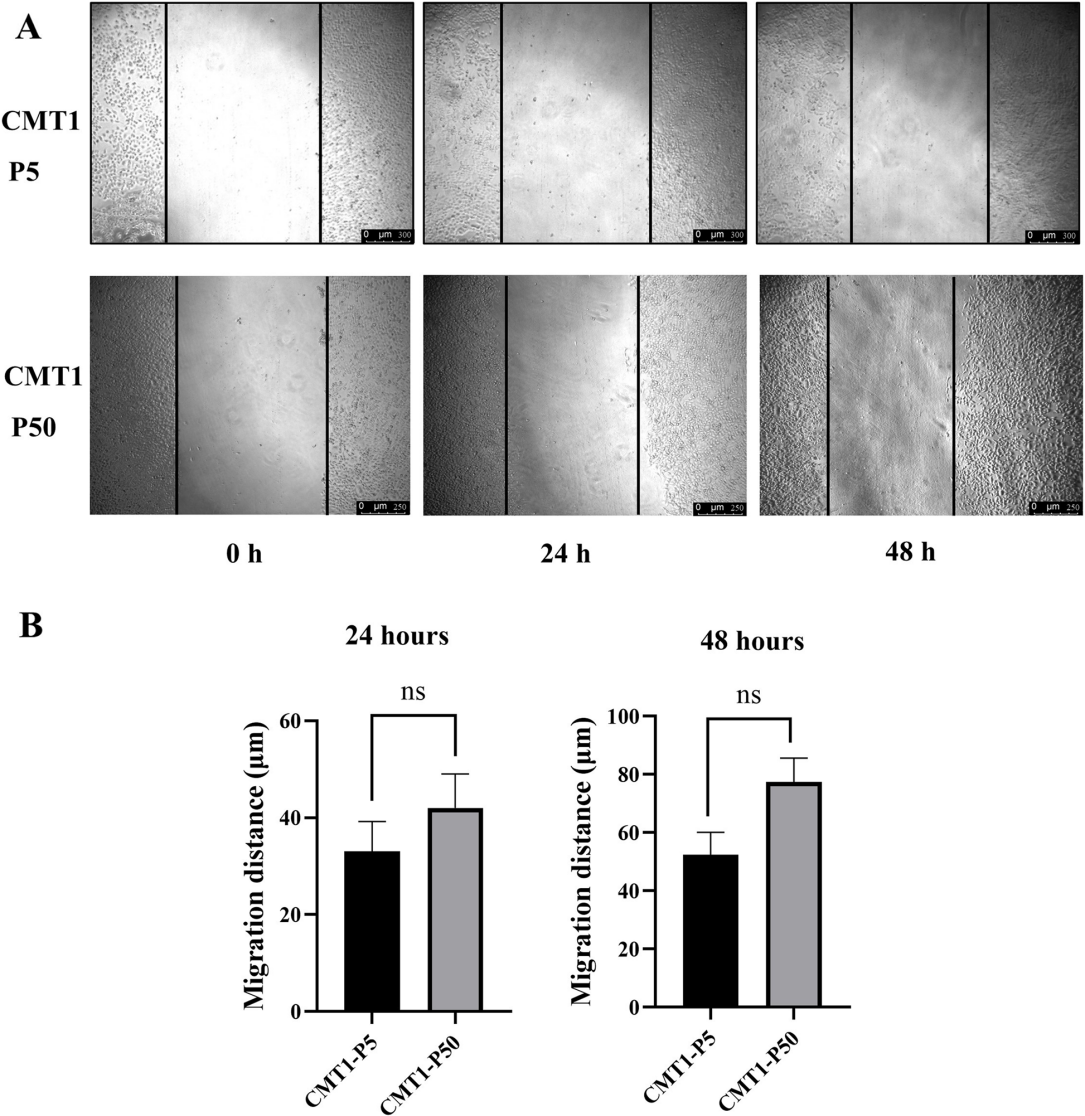
Migration assay. **A** Cell migration ability was tested at 24 and 48 h. The black vertical lines in the images define the open wound areas. **B** The average migration distance of various preparations is shown. All assays were independently repeated at least three times. ns indicates not significant

**Fig. 8:**
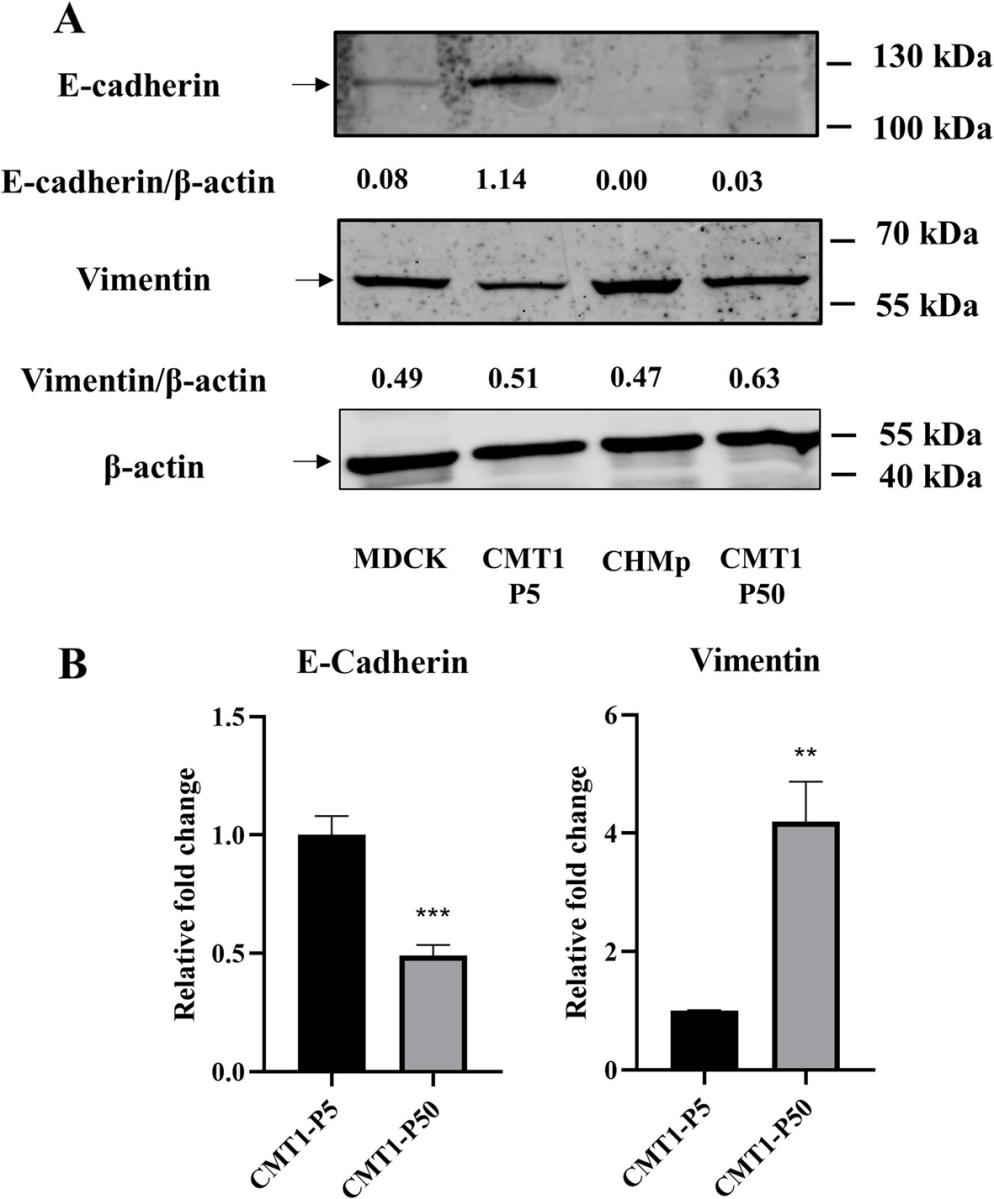
Expression of E-cadherin, and vimentin of CMT-1 cells. **A** WB for E-cadherin and vimentin. MDCK cells showed weak reaction to E-cadherin while CMT-1 cells (passage 5) strongly expressed E-cadherin. CMT-1 cells (passage 50) lost E-cadherin expression but gained greater vimentin expression. **B** Relative fold changes in E-cadherin and vimentin expression using qPCR between CMT1-P5 and CMT1-P50. All experiments were independently repeated at least three times. The data are shown as the mean ± SD, *n* = 3, * *p* < 0.05, ** *p* < 0.01, *** *p* < 0.001). The original images of the western blot were shown in the Supplementary file 1

Our in vivo study showed that CMT-1 cells could induce metastasis; however, no metastasis was detected in the original patient at 14 months post-surgery. In order to assess the abilities of migration rate between cell passages, CMT-1 cells (passage 5 and passage 50) were used for wound healing assays. There is no significant difference in migration distance over 48 h between the two cell passages (Fig. 7). The expression of E-cadherin and vimentin was tested using WB with four different cells. MDCK and CHMp cells were used as the control. CMT-1 cells (passage 5) expressed the highest level of E-cadherin among the four different cells. However, CMT-1 cells (passage 50) showed low expression of E-cadherin, which was consistent with the above-mentioned IFA results. Overtime, CMT-1 cells (passage 50) reduced E-cadherin expression and gained more vimentin expression (Fig. 8A). At the same time, the mRNA expression level of E-cadherin and vimentin in CMT-1 cells (passage 5 and passage 50) were evaluated using qPCR, which showed a statistical significance between the two cell passages (Fig. 8B). The significant difference in E-cadherin and vimentin, the EMT transcriptional factors (EMT-TFs), combined with clinical metastasis in animal experience suggested that CMT-1 cells may undergo potential spontaneous EMT. However, more EMT-TFs such as N-cad, SNAIL1, TWIST1 and ZEB1 need to be assessed to confirm EMT process.

### Morphology of CMT-1 cells on 3D culture

**Fig. 9:**
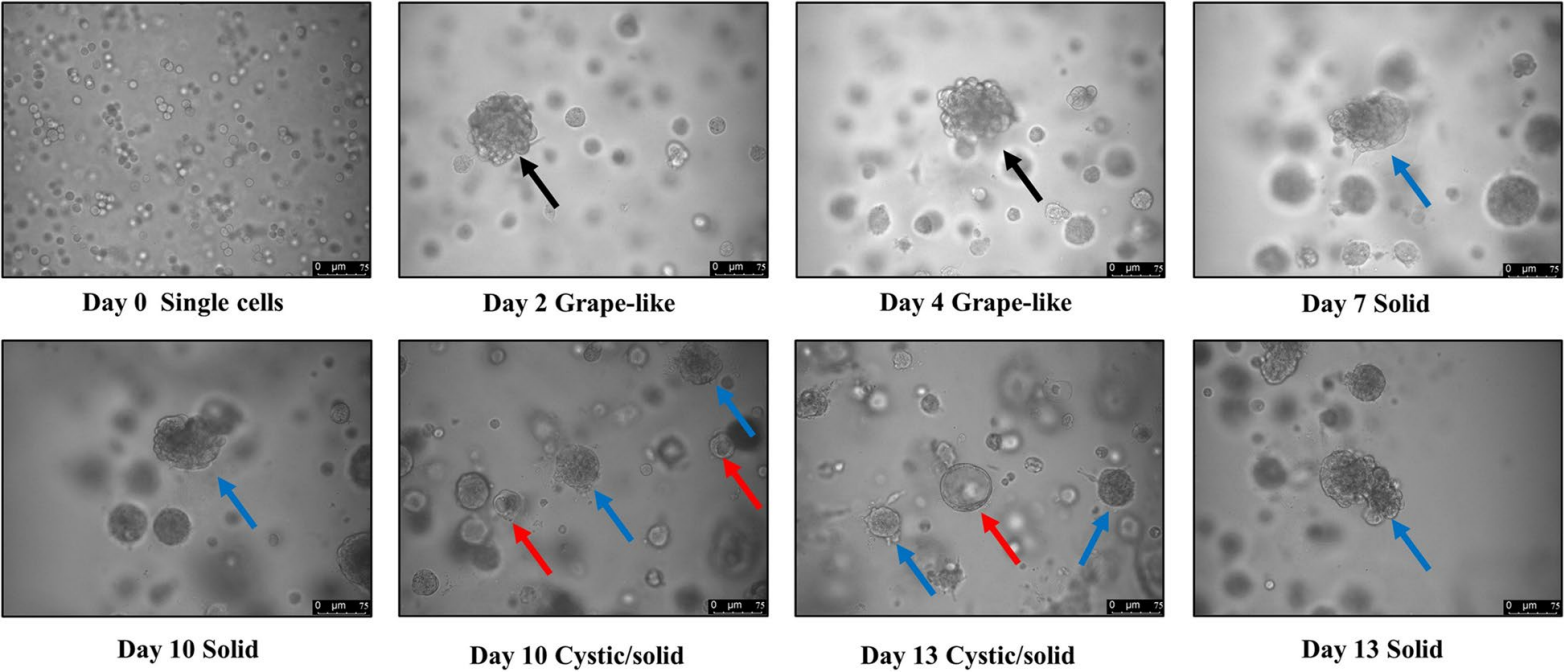
Representative bright-field images of different morphologies of CMT-1 mammary organoids. Black arrow: grape-like, Blue arrow: solid, Red arrow: cystic. Scale bar = 50 μm (indicated in the bottom right corner of the image)

The morphology of CMT-1 cells on 3D culture was assessed. Mammospheres developed from CMT-1 homogeneous cells demonstrated grape-like, solid, cystic, or mixed morphologies (Fig. 9). During mammosphere culture, the morphology of the organoid became more solid and cystic.

### Tumorigenicity of CMT-1 cells in mice

**Fig. 10:**
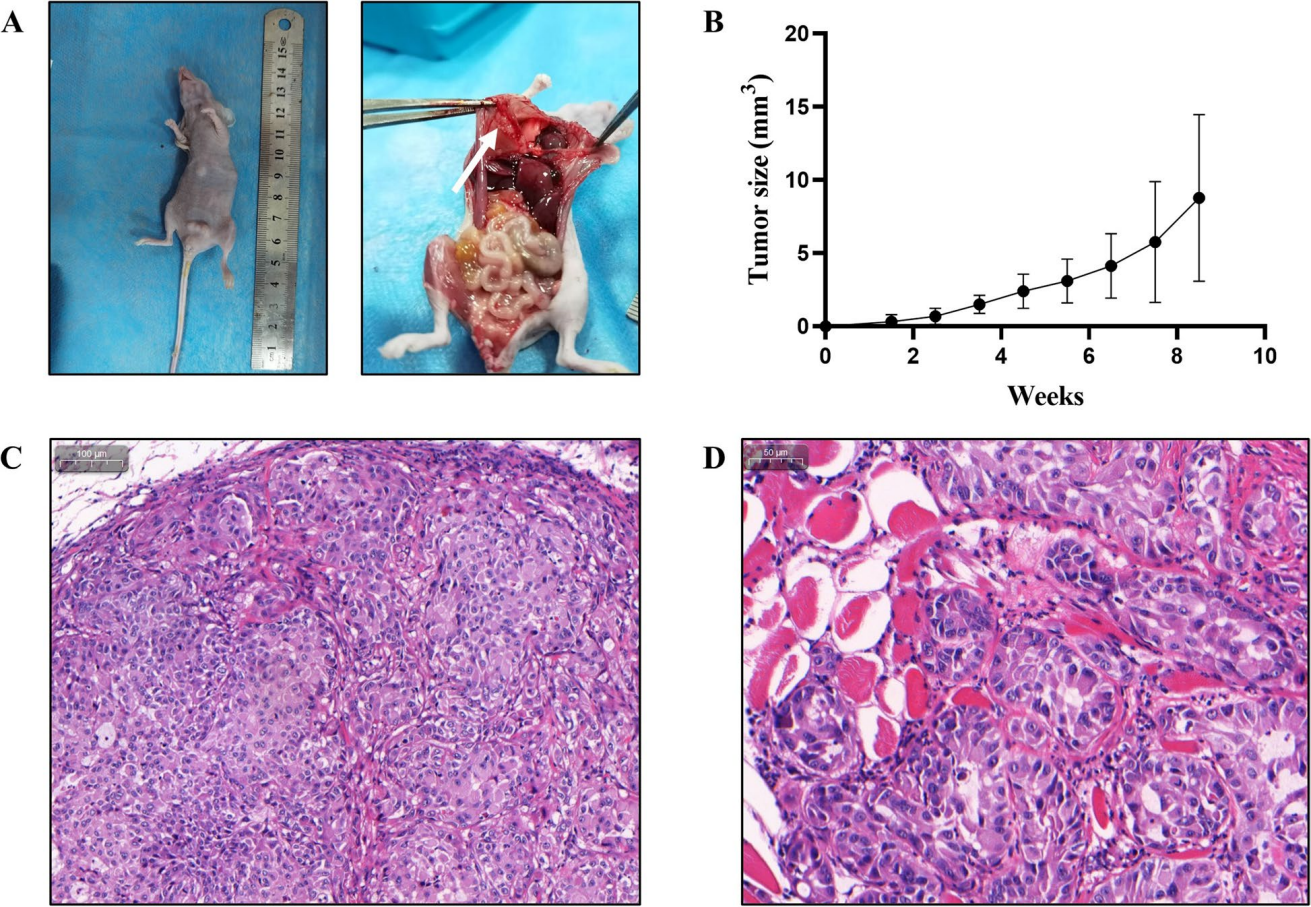
Nude mouse tumorigenicity assay. **A** The primary tumor appeared as a small solid mass at the injection site. Diffuse to coalescing whitish lesions (white arrow) were noted on the diaphragm. **B** Tumor volume of the nude mice. The data are shown as the mean ± SD, *n* = 10, (**C**) Epithelial cells formed nests and irregular tubules on a spindoloid stromal proliferation in a primary xenograft tumor. Scale bar = 100 μm (indicated in the upper left corner of the image). **D** Diaphragmatic muscle degeneration and atrophy adjacent to the neoplastic cell infiltrates. Scale bar = 50 μm (indicated in the upper left corner of the image)

**Fig. 11:**
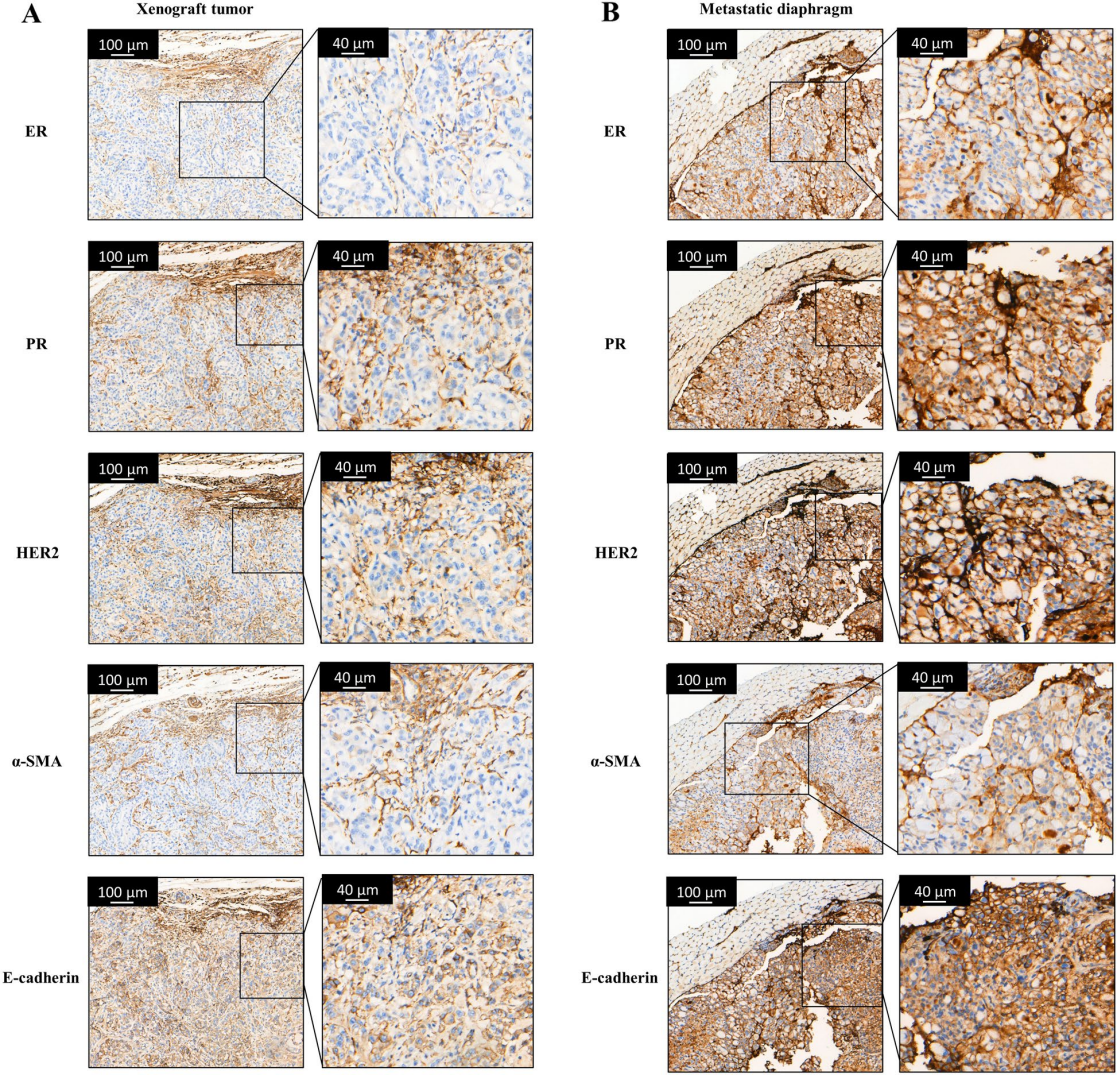
IHC of the xenograft tumor (**A**) and the metastatic diaphragm (**B**). Both the xenograft tumor and metastatic diaphragm were negative for ER and PR, but positive for HER2, α-SMA, and E-cadherin. The metastatic diaphragm had stronger staining for HER2 and α-SMA

In order to evaluate the tumorigenicity of this cell line, CMT-1 cells were injected subcutaneously into the left inguinal mammary fat pad of mice, with the injection bleb being completely absorbed within 1 min. The tumor grew slowly in early stages. Most of the tumors appeared as small bean-like soft solid masses (Fig. 10A). The tumor size varied in mice but the overall size increased throughout the experiment (Fig. 10B). Surprisingly, diaphragmatic metastatic lesions were noticed during necropsy in three mice (Fig. 10A, white arrow). Seven mice were confirmed to have complex mammary adenocarcinoma with no metastasis histologically, whereas three mice were consistent with complex mammary adenocarcinoma with metastatic diaphragmatic adenocarcinoma. The epithelial cells formed tubular structures. The cells had slightly vacuolar to granular cytoplasm with the nuclei being round to oval with vesicular chromatin (Fig. 10C). There was moderate anisocytosis and anisokaryosis. The skeletal muscle of the diaphragm showed degeneration and atrophy with infiltrates of neoplastic cells (Fig. 10D). The immunophenotype markers of the xenograft tumor were similar to those of the canine primary tumor, which was ER and PR negative but HER-2, α-SMA, and E-cadherin positive. (Fig. 11). Interestingly, although the immunophenotype markers of the metastatic lesion were similar to the primary xenograft tumor, it appeared to have stronger HER2 and α-SMA staining. Therefore, we suspected the metastatic tissue was mainly composed of myoepithelial cells. This in vivo experiment demonstrated that the CMT-1 cell line could produce tumors that resembled the original tumor but with greater malignancy both clinically and molecularly.

## Discussion

### Clinical significance of myoepithelium and HER2 expression

The mammary gland consists of luminal epithelial cell lineage and basal ME cell lineage. ME cells are contractile epithelial cells that lie in between the luminal epithelial cells and basement membrane [19, 22]. In addition to cell morphology, the use of cell markers is another way to identify myoepithelium. It is recommended to use multiple biomarkers to identify myoepithelial cells [4]. Currently, biomarkers used for defining myoepithelial cells are α-SMA, p63, calponin, cytokeratins, CD10 and etc. [4, 23, 24]. α-SMA is the ME cell marker of choice in dogs [25]. *p63* gene is tumor suppressor gene under *p53* gene family and p63 protein locates in the nucleus. In our study, CMT-1 cells were positive to α-SMA, p63 and vimentin. Sammarco et al. found malignant myoepithelial cells in feline mammary carcinomas were 100% positive to vimentin but variably positive to p63, cytokeratins and calponin [24]. In the present study, ME cells were successfully isolated from the clinical case and identified based on morphology and molecular expression.

*HER2/ErbB2* is an oncogenic gene associated with tyrosine kinase, facilitating cell proliferation, disruption of cell adhesion and cell polarity, invasiveness, and metastasis [26]. Among all the subtypes of HBC, HER2-positive breast cancer accounted for 11–30% with the HER2-enriched being the most representative subgroup [27]. HER2-enriched subtype tended to metastasize [28]. Therefore, HER2-enriched cell lines are an essential in vitro model for the study of its biological behaviors and to evaluate potential new regimens. Like HBC, CMT was subdivided based on its molecular expressions and each subtype has its own clinical significance that is worth studying. Recently, Kabir et al. determined canine epithelial cell line CMT9, CMT27 and CMT28 were HER2 subtype using qRT-PCR[29]. In current study, CMT-1 cells expressed no nuclear ER and PR, but did express HER2 in WB, IFA, and IHC. Therefore, we determined CMT-1 cell line is a canine HER2-enriched myoepithelial cell line.

In our study, a 14-month follow-up with the patient confirmed no CMT recurrence after routine mastectomy with no adjunctive therapy. It is consistent with the observation that complex mammary carcinoma may have better prognosis than simple mammary carcinoma [30]. However, injecting purified ME cells into nude mice induced a mixed mammary tumor with metastasis. These results are contrary to each other. Over the past two decades, the role of ME cells in mammary carcinogenesis remains controversial. Much evidence has shown that normal ME cells act as “natural tumor suppressors” through paracrine regulation and microenvironmental alteration of the tumor [31, 32]. Nevertheless, Lo et al. found that tumor-associated ME cells may actually promote tumor invasion through the TFG-β signaling pathway [33]. It is also hypothesized that the tumor suppressive effect of myoepithelial cells is dependent on its differential stages [19, 34]. In the present study, ME cells acted as tumor promoters. This phenomenon may be related to the stem cell property/undifferentiated stage of the myoepithelium, increased HER2 expression, and potential spontaneous EMT process. However, further studies need to be conducted to elucidate the role of ME cells in mammary carcinogenesis and hopefully to determine their prognostic value.

### CMT-1 cells experienced potential spontaneous EMT

EMT has a dual nature. In normal development, EMT and its reverse process MET are critical for embryogenesis. However, in cancer, it is associated with tumor stemness, metastasis, and resistance to therapy [35].

E-cadherin is the prototypical cell marker of EMT, expressed in epithelial cells. Vimentin is another classical cell marker of EMT, expressed in mesenchymal cells. During EMT, the expression of E-cadherin decreases while that of vimentin increases [36]. Our results showed that, compared with CMT-1 cells (passage 5), a significant increase in vimentin and decrease in E-cadherin in CMT-1 cells (passage 50) was noted in both qPCR and WB. Moreover, metastatic lesions were found in the diaphragm in our transplantation model. As a result, we suspected CMT-1 cells had undergone a potential spontaneous EMT process where CMT-1 cells (passage 5) lost its epithelium-like phenotype and CMT-1 cells (passage 50) adopted mesenchymal cell characteristics. However, more EMT transcriptional factors such as N-cad, SNAIL1, ZEB1 and TWIST1 need to be confirmed to prove spontaneous EMT process of CMT-1 cells.

### CMT-1 cells formed mammospheres with different morphologies

In this study, we demonstrated canine mammosphers with different morphologies formed by CMT-1 cell line, including grape-like cell clusters, spheroid solid cell balls, and single cystic/luminal structures. With this mammosphere, we may study the embryogenesis of the myoepithelial cell or elaborate the tumorigenesis of CMTs, or specifically malignant myoepithelioma and screen for new drugs to combat tumor resistance in the future.

## Conclusion

In this study, we successfully established a HER2-enriched canine mammary myoepithelial cell line and its associated mammospheres with in vivo and in vitro study. The development of the CMT-1 cell line and mammospheres are valuable for the study of the role of myoepithelial cells in tumorigenesis.

## Materials and methods

### Establishment and purification of primary cultures

Mammary tumor tissues were cut into small pieces (1 ~ 3 mm^3^) and washed three times with phosphate-buffered saline (PBS; Biological Industries, Kibbutz Beit-Haemek, Israel) containing 1% penicillin–streptomycin solution (Beyotime, Shanghai, China). Tumor samples were digested with TrypLE Express (Gibco, Grand Island, NY, USA) for at least 6 h. The digested tissue suspension was collected and placed in a 15 mL centrifuge tube for centrifugation at 1000 rpm for 10 min. The cells at the bottom were resuspended in Dulbecco’s modified Eagle medium (DMEM; Biological Industries) with 20% fetal bovine serum (FBS) (Gibco) and 1% penicillin–streptomycin solution (Beyotime). The cells were placed on 12-well plates and cultivated at 37 °C in a humidified atmosphere containing 5% carbon dioxide (CO_2_). Cells were observed daily by microscopy. When the cell cultures reached 90% confluence, the cells were incubated with TrypLE Express. The digested cells were transferred into new 25 cm^2^ flasks at a density of ∼1 × 10^5^ cells/mL. Cohorts of cells were frozen at every five passages. CMT-1 was considered as an established cell line after passage 50.

### Growth studies and doubling time

CMT-1 cells (passage 50) were cultured on 12 well plates, with 5 × 10^3^ cells per well, and maintained in DMEM supplemented with 10% FBS for 10 d. At 24-h intervals, three replicative wells were digested and counted with Countess 3 (Invitrogen, Carlsbad, CA, USA). Cell growth doubling time was calculated from the exponential growth phase.

### Cytogenetic analysis

CMT-1 cells (passage 55) were seeded into a 6 well cell culture plate and incubated at 37 °C with 5% CO_2_. When the cells reached 60% confluence, they were treated with 0.05 μg/ml colchicine. After being cultured for 6 h at 37 ℃ with 5% CO_2_, the cells were dissociated with TrypLE Express (Gibco, Grand Island, NY, USA), resuspended with a preheated 0.075 mol/L KCL solution and bathed in 37 ℃ water for 1 h. Cells were fixed in methanol‐acetic acid (3:1). Dropped the cell suspension from a height of 1.5 m onto a moist slide precooled at 4 ℃. Stained with Giemsa solution (Beyotime Biotechnology, China) for 15 min, the slides were gently washed with ultrapure water. 100 cells with good chromosome dispersion in metaphase were selected for observation and analysis under the Leica optical microscope and the chromosome number were counted and recorded.

### Wound healing assays

In cell migration assays, CMT-1 cells (passage 5 and passage 50) were seeded on 12-well plates. The cell motility was assessed by measuring the movement of cells into a scarp. The speed of scarp closure was monitored after 12, 24, 36, and 48 h by measuring the ratio of the distance of the scarp from 0 h. Each experiment was performed in triplicate.

### Quantitative real-time PCR (qRT-PCR)

Total RNA was extracted from CMT-1 cells at passage 5 and passage 50 using a Simply P Total RNA Extraction kit (Bioer Technology, Hangzhou, China) according to the manufacturer’s instructions. cDNA samples (1000 ng) were prepared from total RNA using a HiScript III 1st Strand cDNA Synthesis Kit (Vazyme, Nanjing, China). qRT-PCR was carried out on a LightCycler 480 (Roche, Basel, Switzerland) with ChamQ SYBR qPCR Master Mix (Vazyme) using the following program: 95 °C for 30 s, followed by 40 cycles of 95 °C for 10 s, and 60 °C for 30 s. Samples were analyzed in triplicate and three independent experiments were performed. The mRNA expression levels of target genes relative to glyceraldehyde 3-phosphate dehydrogenase (GAPDH) were calculated with the 2^−∆∆Ct^ method and plotted as fold changes compared between cells from passage 5 and passage 50. Primers used for qRT-PCR were designed based on published sequences and are listed in Supplementary file 2 Table S1.

### Western blotting (WB)

CMT-1 (passage 5) and CMT-1 (passage 50) cells were seeded on 6-well plates, respectively. After 100% confluence, the cells were lysed using Cell Lysis Buffer for Western blot and IP (Beyotime). A total of 30 μg of each sample was separated by sodium dodecyl sulfate polyacrylamide gel electrophoresis (SDS-PAGE) and transferred to a nitrocellulose membrane that was blocked for 15 min at room temperature (RT) with QuickBlock Blocking Buffer for Western Blot (Beyotime) and then incubated overnight at 4 °C with primary antibodies, followed by a 1 h incubation with a secondary antibody. Protein bands were visualized by Odyssey Sa (Li-cor, Lincoln, NE, USA). The primary and secondary antibodies used in this study are listed in Supplementary file 2 Table S2.

### Indirect immunofluorescence analysis (IFA)

CMT-1 cells (upon passage 50) were seeded on a 12-well plate with glass slides. The cells were washed three times with PBS and then fixed in cold 4% paraformaldehyde at RT for 10 min. For immunofluorescence staining, cells were blocked for 10 min at RT (QuickBlock Blocking Buffer for Immunol Staining; Beyotime). Then the cells were incubated overnight at 4 °C with the primary antibody and for 1 h at RT with the secondary antibody. The nuclei were stained with DAPI (Beyotime) for 5 min. The fluorescence signal was observed with a confocal laser scanning microscope (SP8 TCS; Leica, Wetzlar, Germany). Primary and secondary antibodies used in this study are listed in Supplementary Table S2.

### Histopathology and immunohistochemistry (IHC)

For histopathological assessment, representative tumors and other tissues with lesions were fixed in 10% buffered formalin, processed by routine methods, embedded in paraffin wax, sectioned at 3 µm and stained with hematoxylin and eosin (HE). Histopathological findings were recorded and used to classify the tumors according to Goldschmidt et al. [20]. For IHC assessment, the tissue samples including CMT-1 induced tumors, lungs, and diaphragm were prepared in 3 µm samples. Tissues were deparaffinized for 20 min and underwent heat-induced antigen retrieval with high pressure-heated methods in sodium citrate buffer (pH 6.0) for 3 min, then cooled to RT. The slides were covered with 3% hydrogen peroxide for 10 min to terminate the peroxidase activity. Specimens were then incubated with primary antibodies (listed in Supplementary Table S2) at 37 °C for 60 min and with secondary antibody (BOND Polymer Refine Detection; Leica Biosystem, Buffalo Grove, IL, USA) at 37 °C for 1 h in order to detect target proteins. Each specimen was washed three times with PBS. Signal was visualized using 3,3 ′-diaminobenzidine (DAB). The samples were counterstained with hematoxylin, dehydrated, cleared, and mounted. Interpretation of HER2 staining was performed according to the Herceptest™ interpretation manual.

### Transplantation into mice

For tumorigenicity assays, a randomized analytic experiment was performed. Fifteen 8-week-old female BALB/c nude mice (Vital River Laboratory Animal Technology, Beijing, China) with a mean body weight of 20.06 g were included. The study was carried out in compliance with the ARRIVE guidelines and was approved by the Institutional Animal Care and Use Committee of South China Agricultural University (Permit Number: 2021B102). All mice were randomly separated into groups A, B, and C. Each group contained five mice. A 0.2 mL DMEM suspension of CMT-1 (passage 50) containing 1 × 10^7^ cells was subcutaneously inoculated into the left inguinal mammary fat pads of mice in group A and B. Mice in group C were injected with 0.2 mL saline per mouse at the same location, as the negative control. The growth of tumors was monitored and measured weekly and the tumor size was calculated using an equation V = 1/2 × L x W^2^ (V = volume, L = length, W = width). Mice were sacrificed by cervical dislocation at 61 d after inoculation based on American Veterinary Medical Association Guidelines for the Euthanasia of Animals: 2020 Edition. The tumor masses and organs were collected. Neoplasms and organs were collected in 4% paraformaldehyde for histopathological detection and IHC.

### Three-dimension culture

The digested and single cells of CMT-1 cells (passage 50) were resuspended in cold Cultrex Reduced Growth Factor Basement Membrane Extract (Type 2, Select; Bio-Techne, Minneapolis, MN, USA) and seeded in a prewarmed 24-well plate (at 25 μL drops per well). The drops were solidified in a 37 °C and 5% CO_2_ incubator for 30 min, and then 0.5 mL of organoid culture medium (Accuroid medium; Accurate International, Guangzhou, China) was added to each well and refreshed every 3 d.

### Statistical analysis

Relative expression levels are presented as mean ± standard deviation. The statistical significance of differences was evaluated with the unpaired Student’s t-test using Prism v6.0 software (GraphPad, La Jolla, CA, USA) (mean ± standard deviation (SD), * *p* < 0.05, ** *p* < 0.01, *** *p* < 0.001).

## Supplementary Information

**Additional file 1. **Original images of western blots.

**Additional file 2: Table S1. **Primer pairs used for quantitative RT-PCR analysis; **Table S2** Antibody used in this study.

## Data Availability

All datasets supporting the conclusions of this article are during this study are included in this published article and its additional files. The datasets analyzed during the current study. are available from the corresponding author on reasonable request.

## Abbreviations

CMTs: Canine mammary tumors,
ER: Estrogen receptors
PR: Progesterone receptors
HER2: Human epidermal growth receptor-2
EMT: Epithelial to mesenchymal transition
IHC: Immunohistochemistry
HBC: Human breast cancer
EGFR: Epidermal growth factor receptor
ME: Myoepithelial
α-SMA: α-Smooth muscle actin
CSCs: Cancer stem cells
WB: Western blotting
IFA: Immunofluorescence assay
GAPDH: Glyceraldehyde 3-phosphate dehydrogenase

## References

[1] Salas Y, Marquez A, Diaz D, Romero L (2015). Epidemiological Study of Mammary Tumors in Female Dogs Diagnosed during the Period 2002–2012: A Growing Animal Health Problem. PLoS One.

[2] Beveridge WI, Sobin LH (1976). International histological classification of tumours of domestic animals: Introduction. Bull World Health Organ.

[3] Misdorp WER, Hellmen E, Lipscomb TP (1999). Histologic Classification of Mammary Tumors of the Dog and the Cat, 2nd ser.

[4] Pena L, Gama A, Goldschmidt MH, Abadie J, Benazzi C, Castagnaro M, Diez L, Gartner F, Hellmen E, Kiupel M (2014). Canine mammary tumors: a review and consensus of standard guidelines on epithelial and myoepithelial phenotype markers, HER2, and hormone receptor assessment using immunohistochemistry. Vet Pathol.

[5] Sassi F, Benazzi C, Castellani G, Sarli G (2010). Molecular-based tumour subtypes of canine mammary carcinomas assessed by immunohistochemistry. BMC Vet Res.

[6] Kaszak I, Ruszczak A, Kanafa S, Kacprzak K, Krol M, Jurka P (2018). Current biomarkers of canine mammary tumors. Acta Vet Scand.

[7] Perou CMST, Eisen MB, van de Rijn M, Jeffrey SS, Rees CA, Pollack JR, Ross DT, Johnsen H, Akslen LA, Fluge O, Pergamenschikov A, Williams C, Zhu SX, Lønning PE, Børresen-Dale AL, Brown PO, Botstein D (2000). Molecular portraits of human breast tumours. Nature.

[8] Abadie J, Nguyen F, Loussouarn D, Pena L, Gama A, Rieder N, Belousov A, Bemelmans I, Jaillardon L, Ibisch C (2018). Canine invasive mammary carcinomas as models of human breast cancer. Part 2: immunophenotypes and prognostic significance. Breast Cancer Res Treat.

[9] Gama A, Alves A, Schmitt F (2008). Identification of molecular phenotypes in canine mammary carcinomas with clinical implications: application of the human classification. Virchows Arch.

[10] Burrai GP, Tanca A, De Miglio MR, Abbondio M, Pisanu S, Polinas M, Pirino S, Mohammed SI, Uzzau S, Addis MF (2015). Investigation of HER2 expression in canine mammary tumors by antibody-based, transcriptomic and mass spectrometry analysis: is the dog a suitable animal model for human breast cancer?. Tumour Biol.

[11] Liu Y, Chen L, Jiang D, Luan L, Huang J, Hou Y, Xu C (2021). HER2 promotes epithelial-mesenchymal transition through regulating osteopontin in gastric cancer. Pathol Res Pract.

[12] Yarden Y (2001). Biology of HER2 and its importance in breast cancer. Oncology.

[13] Cheang MC, Chia SK, Voduc D, Gao D, Leung S, Snider J, Watson M, Davies S, Bernard PS, Parker JS (2009). Ki67 index, HER2 status, and prognosis of patients with luminal B breast cancer. J Natl Cancer Inst.

[14] Dutra AP, Granja NV, Schmitt FC, Cassali GD (2004). c-erbB-2 expression and nuclear pleomorphism in canine mammary tumors. Braz J Med Biol Res.

[15] Muhammadnejad A, Keyhani E, Mortazavi P, Behjati F, Haghdoost IS (2012). Overexpression of her-2/neu in malignant mammary tumors; translation of clinicopathological features from dog to human. Asian Pac J Cancer Prev.

[16] Araujo MR, Campos LC, Damasceno KA, Gamba CO, Ferreira E, Cassali GD (2016). HER-2, EGFR, Cox-2 and Ki67 expression in lymph node metastasis of canine mammary carcinomas: Association with clinical-pathological parameters and overall survival. Res Vet Sci.

[17] Campos LC, Silva JO, Santos FS, Araujo MR, Lavalle GE, Ferreira E, Cassali GD (2015). Prognostic significance of tissue and serum HER2 and MUC1 in canine mammary cancer. J Vet Diagn Invest.

[18] Moumen M, Chiche A, Cagnet S, Petit V, Raymond K, Faraldo MM, Deugnier MA, Glukhova MA (2011). The mammary myoepithelial cell. Int J Dev Biol.

[19] Gudjonsson T, Adriance MC, Sternlicht MD, Petersen OW, Bissell MJ (2005). Myoepithelial cells: their origin and function in breast morphogenesis and neoplasia. J Mammary Gland Biol Neoplasia.

[20] Goldschmidt M, Pena L, Rasotto R, Zappulli V (2011). Classification and grading of canine mammary tumors. Vet Pathol.

[21] Welsh AW, Lannin DR, Young GS, Sherman ME, Figueroa JD, Henry NL, Ryden L, Kim C, Love RR, Schiff R (2012). Cytoplasmic estrogen receptor in breast cancer. Clin Cancer Res.

[22] Gudjonsson T, Villadsen R, Nielsen HL, Ronnov-Jessen L, Bissell MJ, Petersen OW (2002). Isolation, immortalization, and characterization of a human breast epithelial cell line with stem cell properties. Genes Dev.

[23] Lopuszynski W, Szczubial M, Millan Y, Guil-Luna S, Sanchez-Cespedes R (2019). Martin de Las Mulas J, Smiech A, Bulak K: **Immunohistochemical expression of p63 protein and calponin in canine mammary tumours**. Res Vet Sci.

[24] Sammarco A, Finesso G, Zanetti R, Ferro S, Rasotto R, Caliari D, Goldschmidt MH, Orvieto E, Castagnaro M, Cavicchioli L (2020). Biphasic Feline Mammary Carcinomas Including Carcinoma and Malignant Myoepithelioma. Vet Pathol.

[25] Sanchez-Cespedes R, Millan Y, Guil-Luna S, Reymundo C (2016). Espinosa de Los Monteros A, Martin de Las Mulas J: **Myoepithelial cells in canine mammary tumours**. Vet J.

[26] Kennecke H, Yerushalmi R, Woods R, Cheang MC, Voduc D, Speers CH, Nielsen TO, Gelmon K (2010). Metastatic behavior of breast cancer subtypes. J Clin Oncol.

[27] Raposo LR, Roma-Rodrigues C, Faisca P, Alves M, Henriques J, Carvalheiro MC, Corvo ML, Baptista PV, Pombeiro AJ, Fernandes AR (2017). Immortalization and characterization of a new canine mammary tumour cell line FR37-CMT. Vet Comp Oncol.

[28] Cronin KA, Harlan LC, Dodd KW, Abrams JS, Ballard-Barbash R (2010). Population-based estimate of the prevalence of HER-2 positive breast cancer tumors for early stage patients in the US. Cancer Invest.

[29] Kabir FML, DeInnocentes P, Agarwal P, Mill CP, RieseNd DJ, Bird RC (2017). Estrogen receptor-alpha, progesterone receptor, and c-erbB/HER-family receptor mRNA detection and phenotype analysis in spontaneous canine models of breast cancer. J Vet Sci.

[30] Rasotto R, Berlato D, Goldschmidt MH, Zappulli V (2017). Prognostic Significance of Canine Mammary Tumor Histologic Subtypes: An Observational Cohort Study of 229 Cases. Vet Pathol.

[31] Farhanji B, Latifpour M, Alizadeh AM, Khodayari H, Khodayari S, Khaniki M, Ghasempour S (2015). Tumor suppression effects of myoepithelial cells on mice breast cancer. Eur J Pharmacol.

[32] Duivenvoorden HM, Brockwell NK, Nowell CJ, Simpson KJ, Parker BS (2021). High-content siRNA 3D co-cultures to identify myoepithelial cell-derived breast cancer suppressor proteins. Sci Data.

[33] Lo PK, Zhang Y, Yao Y, Wolfson B, Yu J, Han SY, Duru N, Zhou Q (2017). Tumor-associated myoepithelial cells promote the invasive progression of ductal carcinoma in situ through activation of TGFbeta signaling. J Biol Chem.

[34] Ingthorsson S, Hilmarsdottir B, Kricker J, Magnusson MK, Gudjonsson T (2015). Context-Dependent Function of Myoepithelial Cells in Breast Morphogenesis and Neoplasia. Curr Mol Biol Rep.

[35] Brabletz S, Schuhwerk H, Brabletz T, Stemmler MP (2021). Dynamic EMT: a multi-tool for tumor progression. EMBO J.

[36] Zeisberg M, Neilson EG (2009). Biomarkers for epithelial-mesenchymal transitions. J Clin Invest.

