# Supplementary material for: Establishment and characterization of a HER2-enriched canine mammary cancerous myoepithelial cell line
Source: BMC Vet Res. 2023 Jan 30;19:22. doi: 10.1186/s12917-023-03573-9 (PMC9885638; doi:10.1186/s12917-023-03573-9)
Supplement: Supplementary file 1 — Additional file 1. Original images of western blots. [file 12917_2023_3573_MOESM1_ESM.docx]

**Uncropped blots**

**Figure 5**


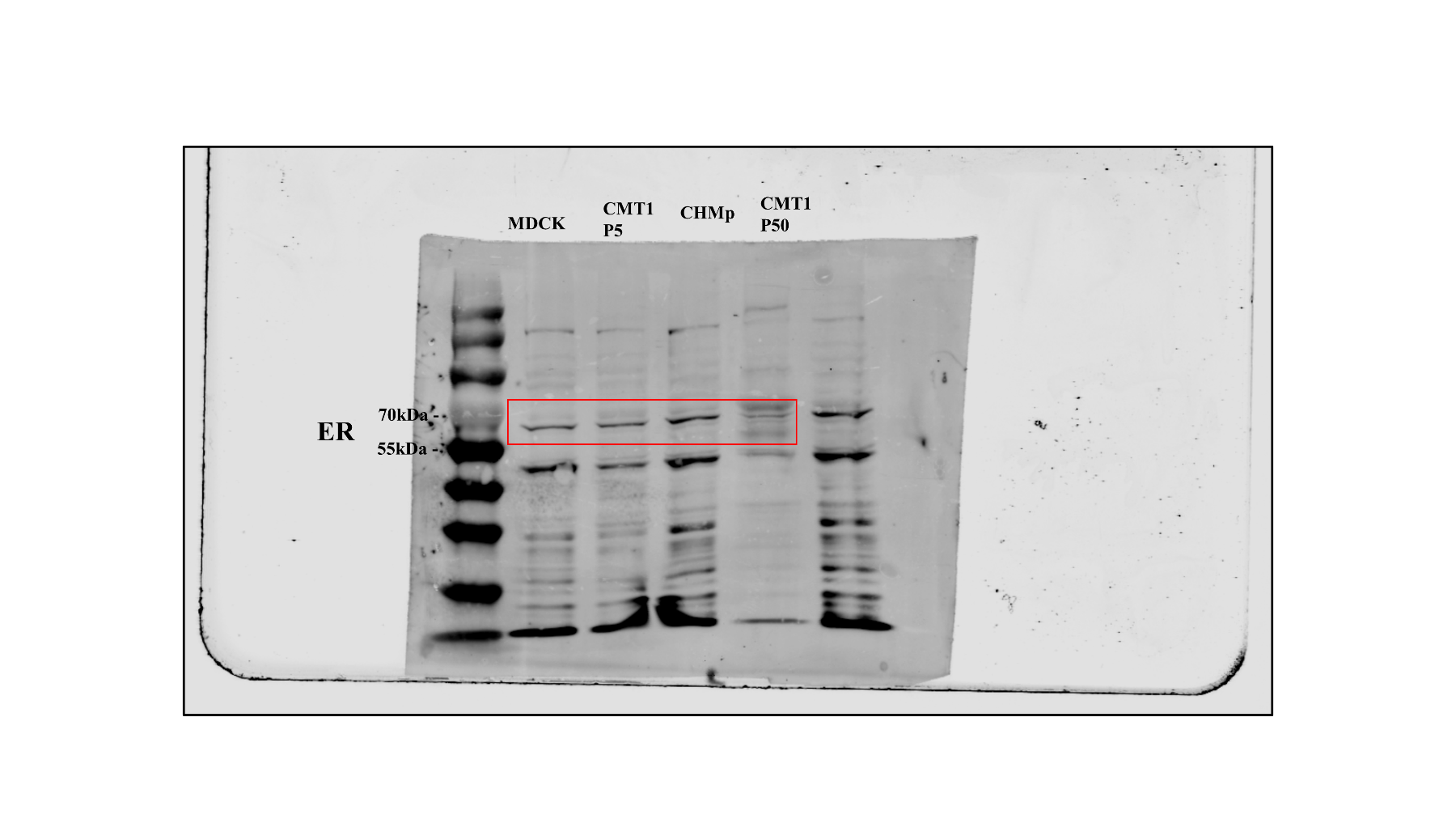


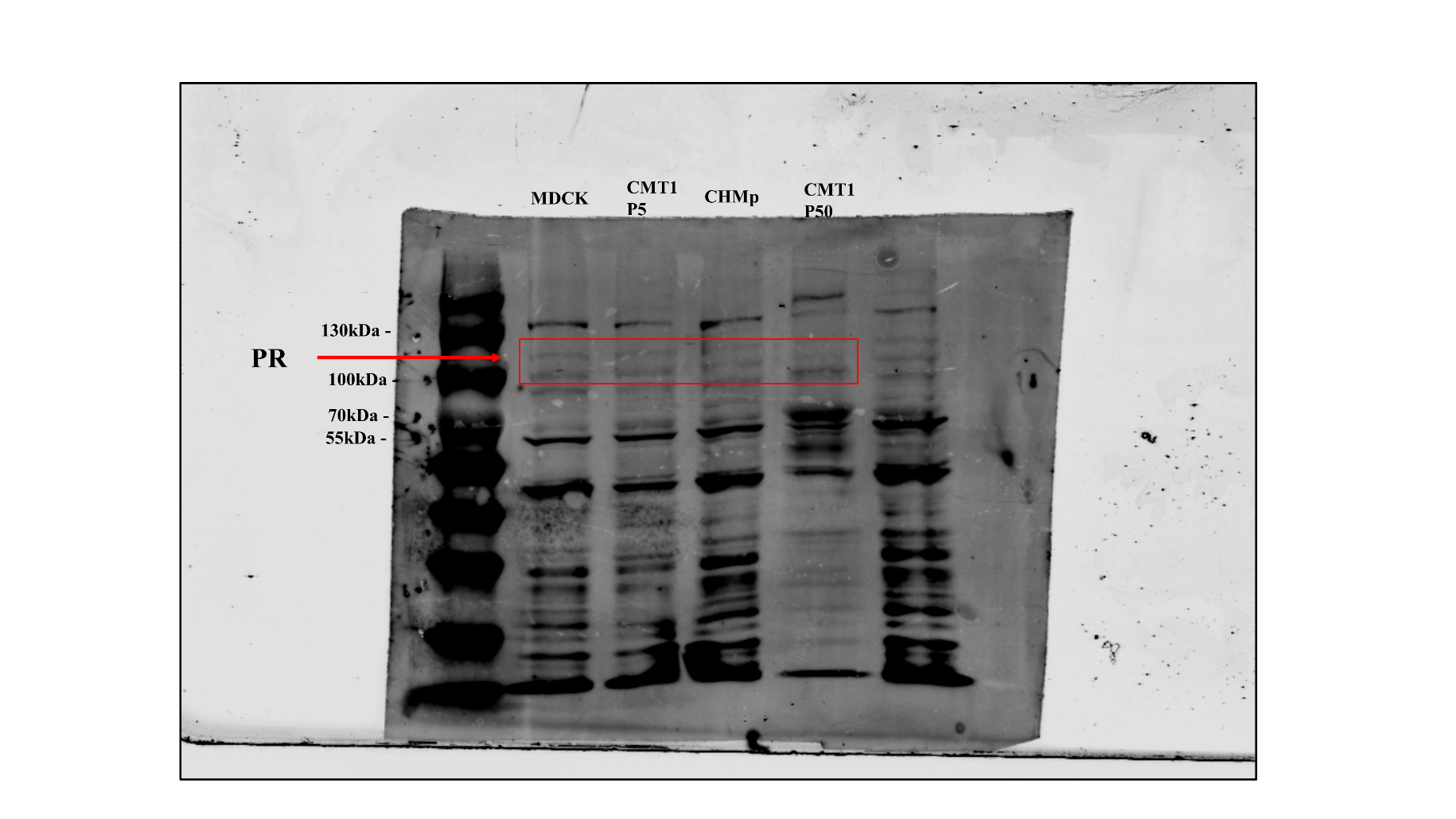


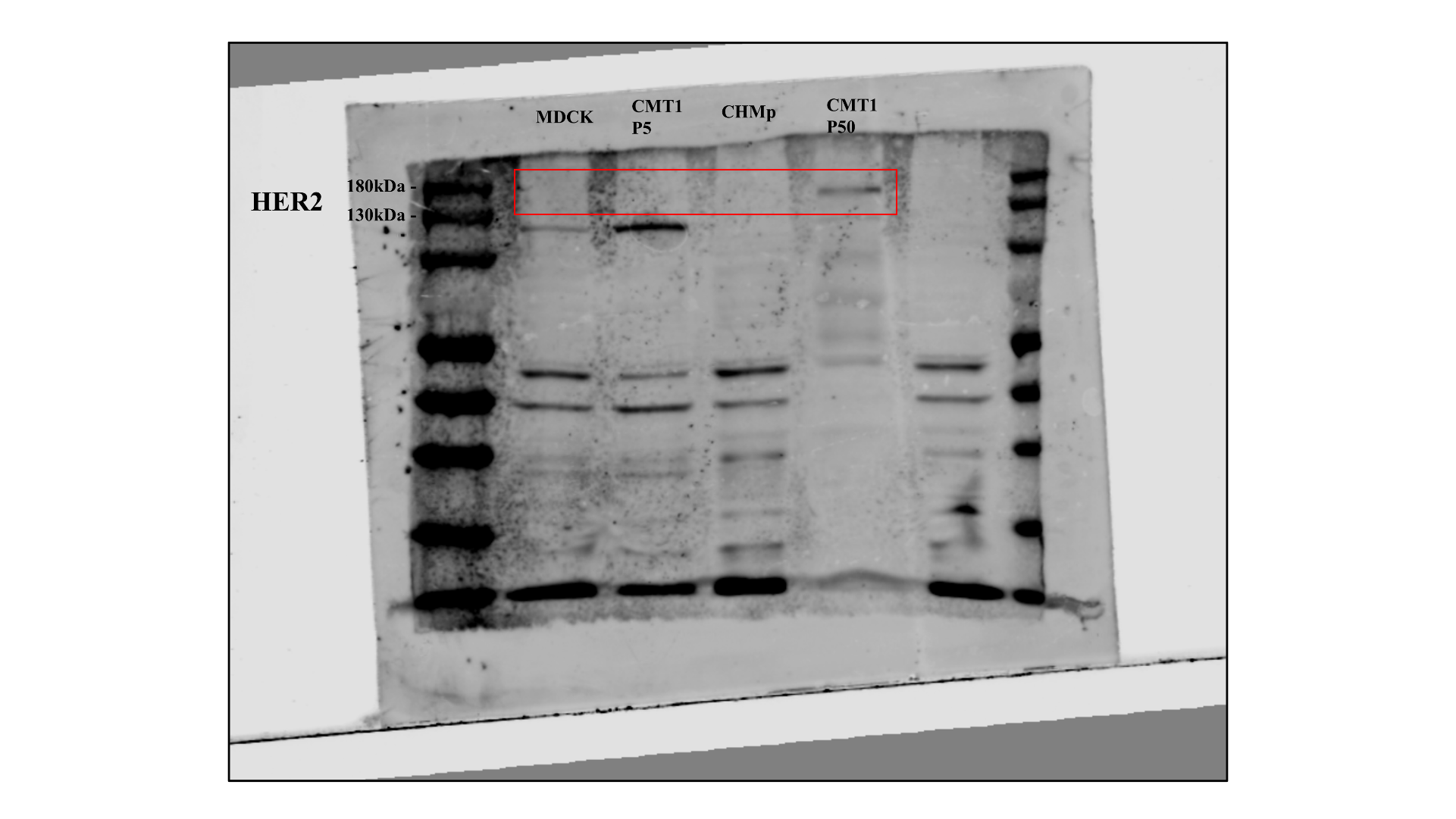

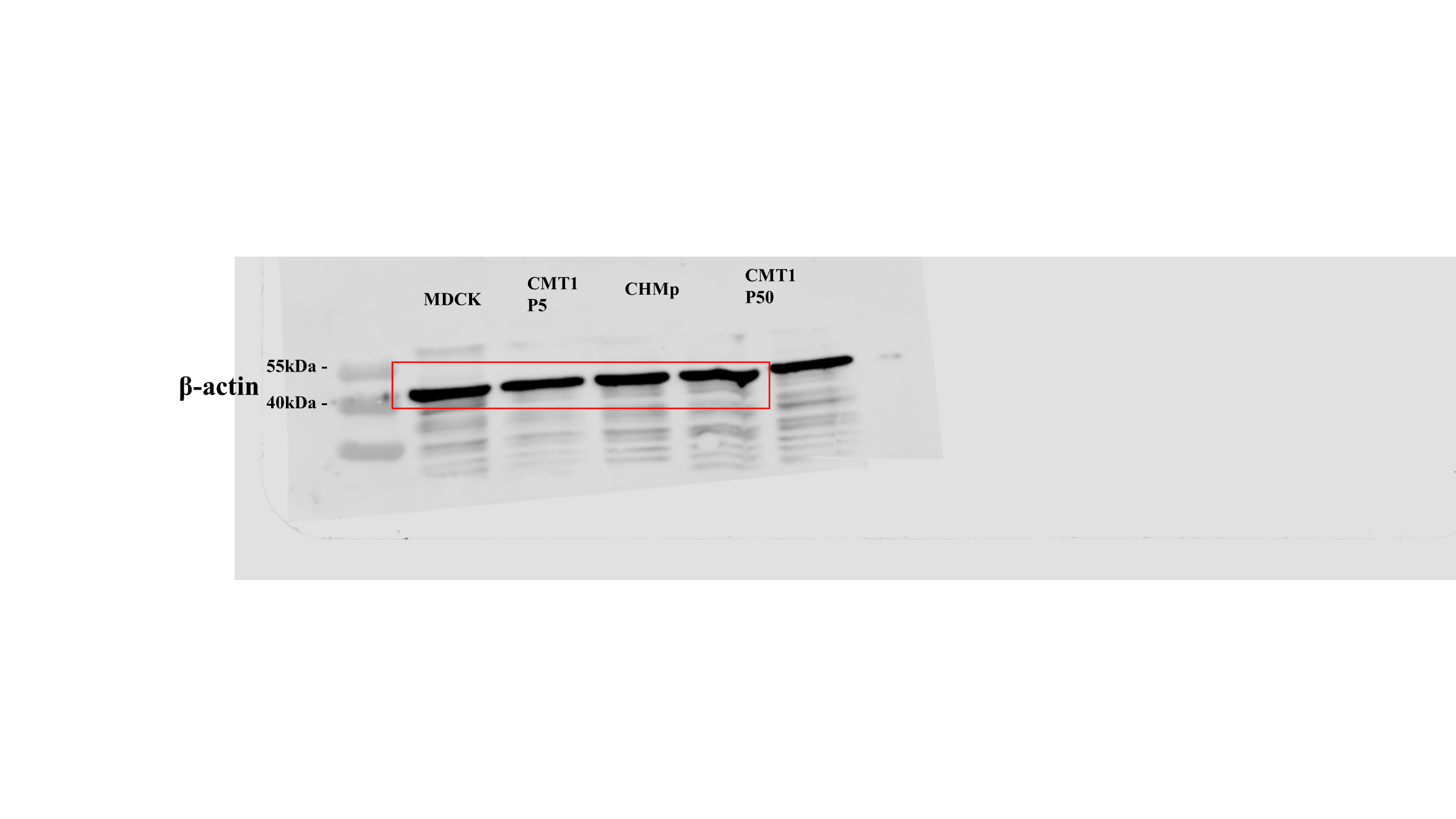


**Figure 5. Western blotting results of different cell lines and immunofluorescence assay results of CMT-1 cells.** (A) The results of western blotting of MDCK cells, CMT-1 cells (passage 5), CHMp cell line, and CMT-1 cells (passage 50) were ER^+^/PR^-^/HER2^-^, ER^+^/PR^-^/HER2^-^, ER^+^/PR^-^/HER2^-^ and ER^-^/PR^-^/HER2^+^ respectively. (B) IFA results of CMT-1 cells were consistent with WB results.

**Figure 6A**


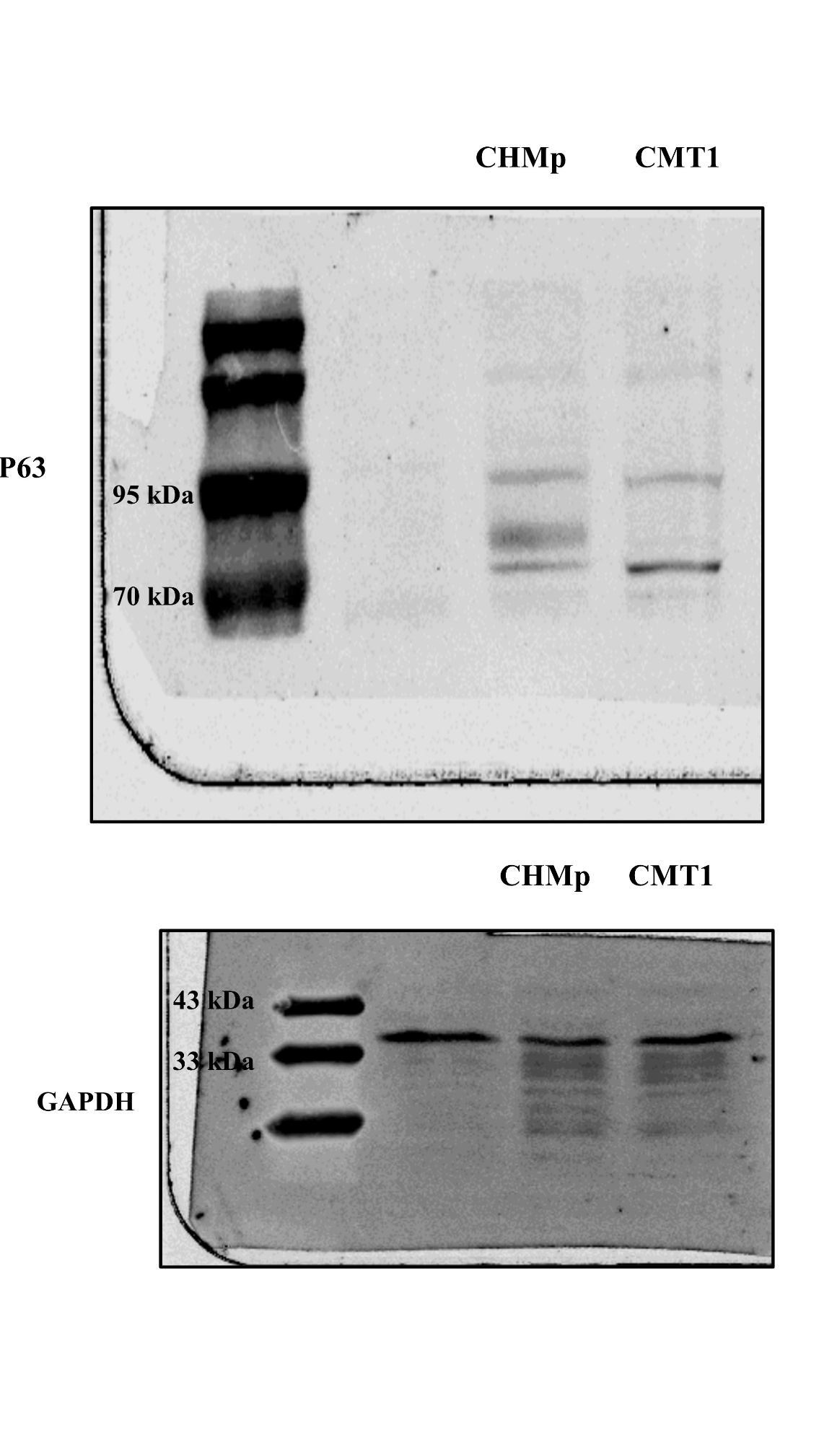


**Figure 6. Molecular analysis of the CMT-1 cell line.** (A&B) The results of western blotting showed CMT-1 cells were positive to α-SMA and p63. (C) IFA results demonstrated CMT-1 cells had weak reaction to E-cad and moderate to strong reaction to vimentin. IFA results shared the same immunoreactivity to α-SMA and p63 for CMT-1 cells.

**Figure 6B**

**
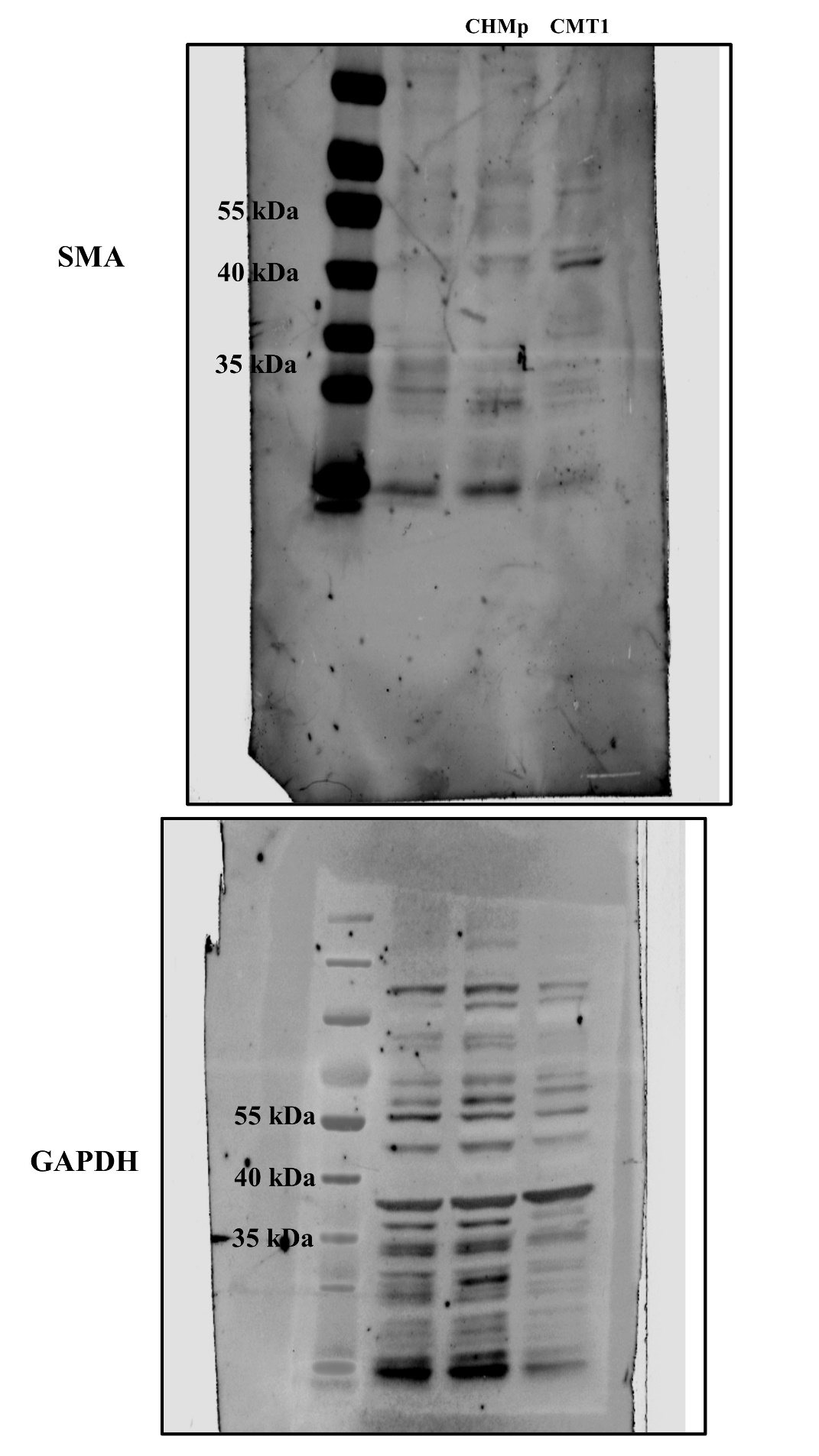
**

**Figure 8A**


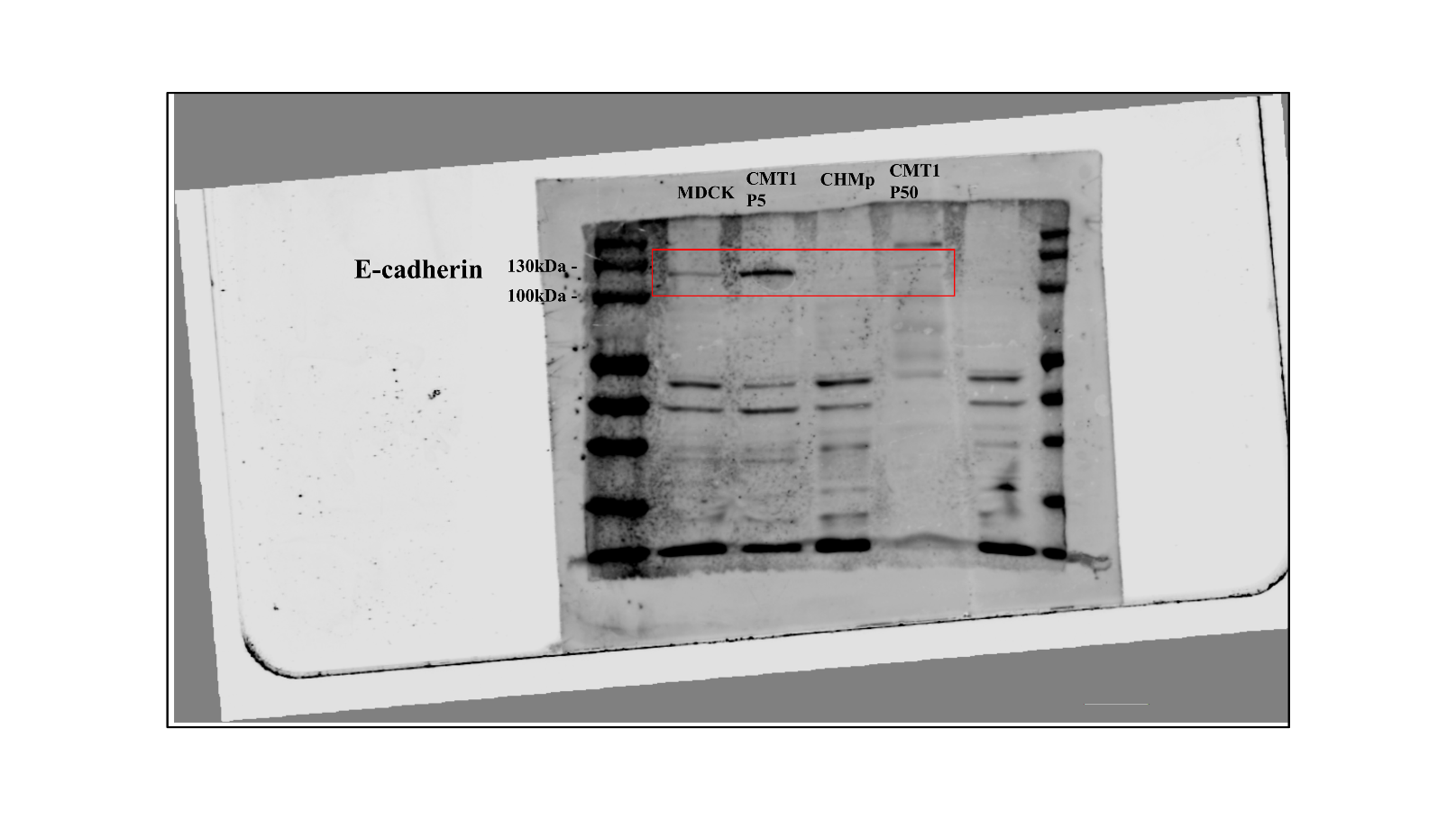

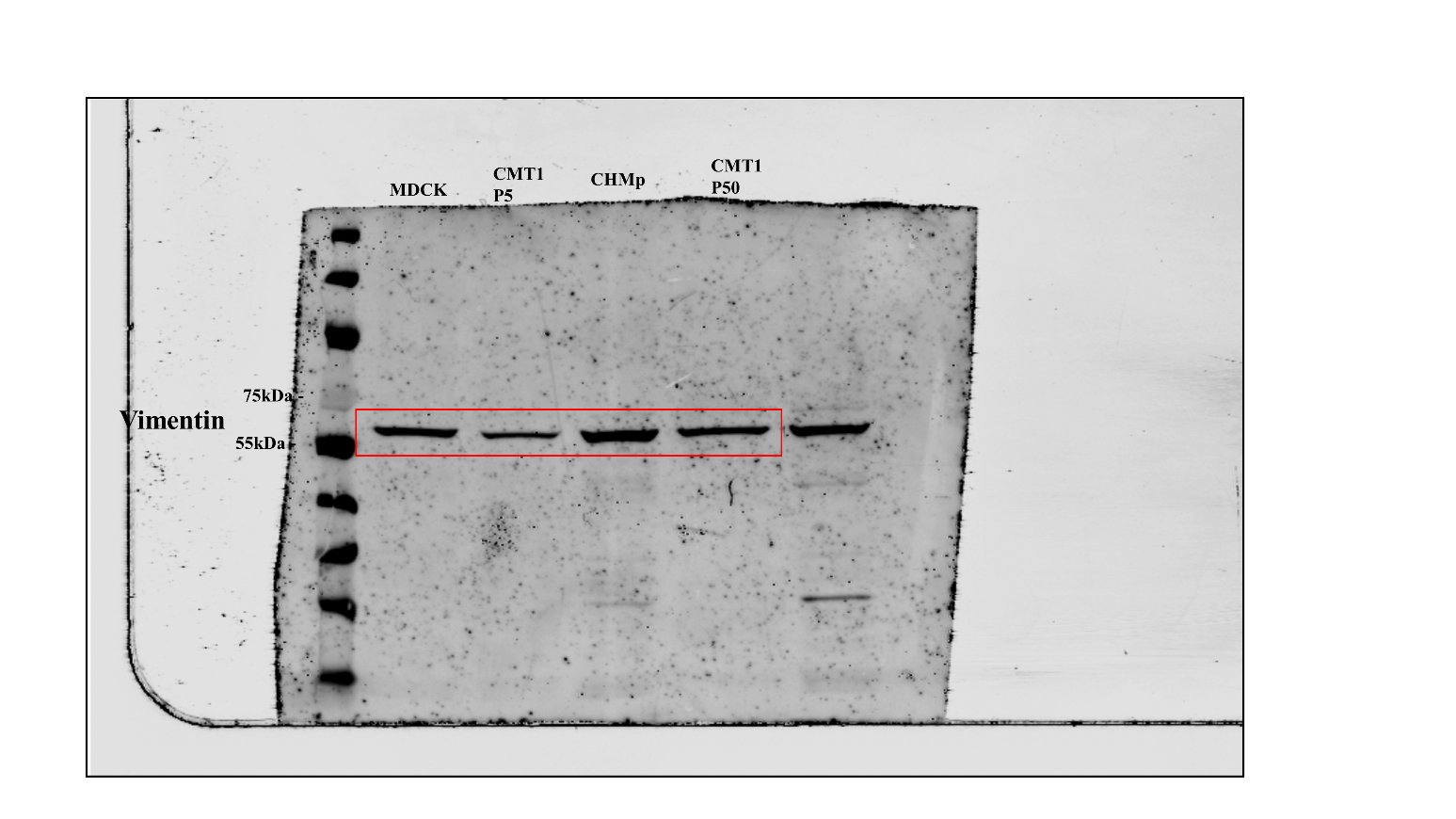


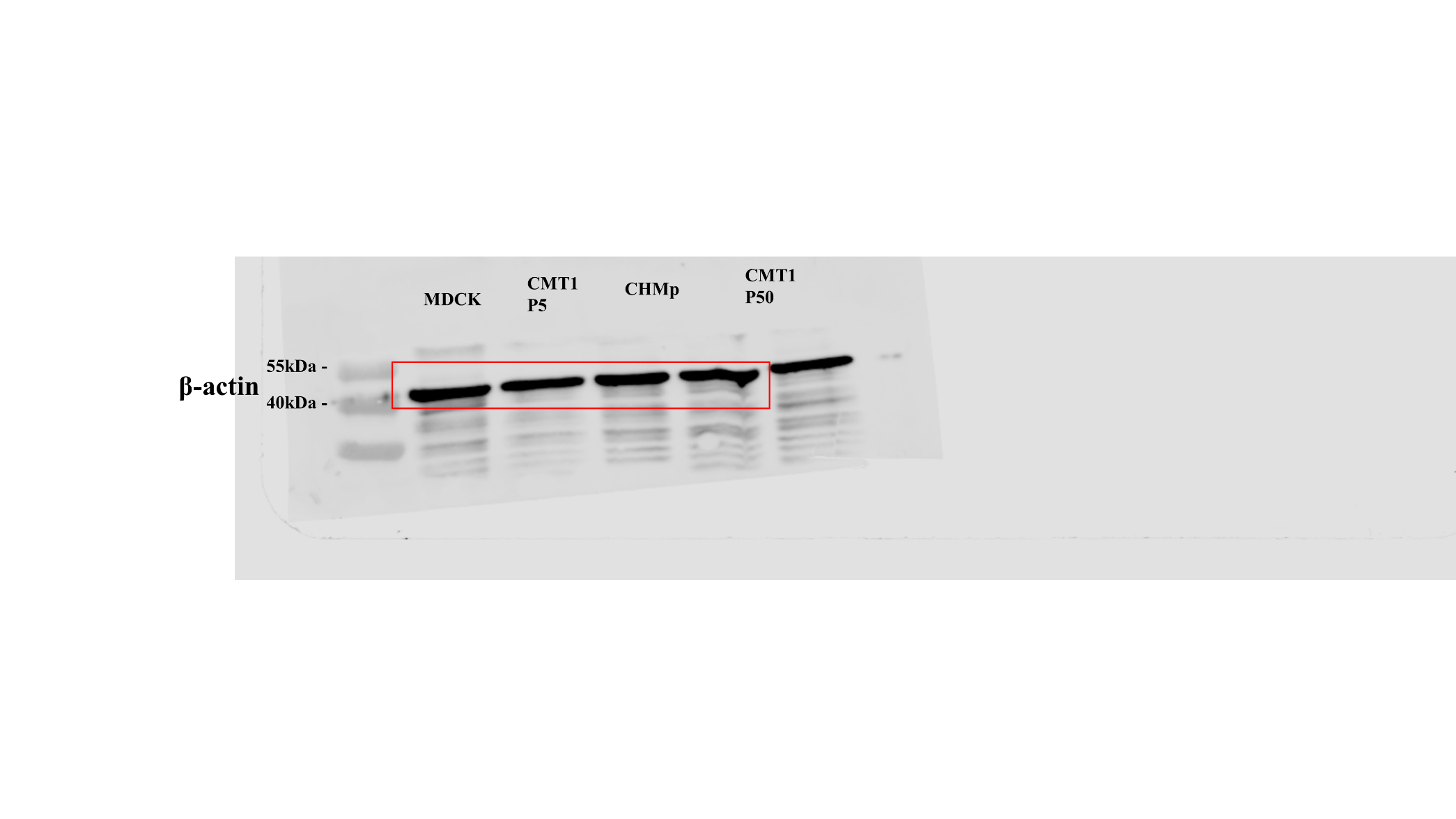


**Figure 8. Expression of E-cad, and vimentin of CMT1 cells.** (A) WB for E-cadherin and vimentin. MDCK cells showed weak reaction to E-cadherin while CMT-1 cells (passage 5) strongly expressed E-cadherin. CMT-1 cells (passage 50) lost E-cad expression but gained greater vimentin expression. (B) Relative fold changes in E-cadherin and vimentin expression using qPCR between CMT1-P5 and CMT1-P50. All experiments were independently repeated at least three times. The data are shown as the mean ±SD, n = 3, * *p* < 0.05, ** *p* < 0.01, *** *p* < 0.001).
