# Supplementary material for: Establishment and characterization of a HER2-enriched canine mammary cancerous myoepithelial cell line
Source: BMC Vet Res. 2023 Jan 30;19:22. doi: 10.1186/s12917-023-03573-9 (PMC9885638; doi:10.1186/s12917-023-03573-9)
Supplement: Supplementary file 2 — Additional file 2: Table S1. Primer pairs used for quantitative RT-PCR analysis; Table S2 Antibody used in this study. [file 12917_2023_3573_MOESM2_ESM.docx]

**Table S1** Primer pairs used for quantitative RT-PCR analysis

| **Primer** | **Sequence of oligonucleotides (5′ → 3)** | **Gene accession NO.** |
| --- | --- | --- |
| E-cadherin-qF | AAATCACATCCTACACCGCC | NM_001287125 |
| E-cadherin-qR | ATTAACCTCCAGCCAACCG |  |
| Vimentin-qF | ACAATGCTTCTTTGGCACGTCT | NM_001287023 |
| Vimentin -qR | TACCATTCCTCGGCCTCCTGA |  |
| GAPDH-qF | AATTCCACGGCACAGTCAAGGC | NM_001003142 |
| GAPDH-qR | ACAACATACTCAGCACCAGCATCAC |  |

**Table S2** Antibody used in this study

| **Antibody** | **Company** | **Host species** | **Catalog number** | **Used for** |
| --- | --- | --- | --- | --- |
| ER | MXB® Biotechnologies | Rabbit | SP1 | IHC |
| PR | MXB® Biotechnologies | Rabbit | IE2 | IHC |
| HER2 | MXB® Biotechnologies | Rabbit | EP3 | IHC |
| E-cadherin | MXB® Biotechnologies | Mouse | 4A2C7 | IHC |
| Vimentin | GeneTech Company | Mouse | V9 | IHC |
| α-SMA | MXB® Biotechnologies | Mouse | MX097 | IHC |
| β-actin | GenteTex | Rabbit | GTX109639 | WB |
| ER | Abcam | Rabbit | AB32063 | IFA and WB |
| PR | Abcam | Rabbit | AB32085 | IFA and WB |
| HER2 | Abcam | Rabbit | AB32085 | IFA and WB |
| E-cadherin | GenteTex | Rabbit | GTX135001 | IFA and WB |
| Vimentin | GenteTex | Rabbit | GTX135083 | IFA and WB |
| α-SMA | ABclonal | Rabbit | A17910 | IFA and WB |
| p63 | Baijia | Rabbit | IPB5265 | IFA and WB |
| Alexa Fluor® 488 | Abcam | Goat | AB150077 | IFA |
| Alexa Fluor® 680 | Abcam | Goat | AB175773 | WB |
